# Biotic and abiotic factors investigated in two *Drosophila* species – evidence of both negative and positive effects of interactions on performance

**DOI:** 10.1038/srep40132

**Published:** 2017-01-06

**Authors:** Michael Ørsted, Mads Fristrup Schou, Torsten Nygaard Kristensen

**Affiliations:** 1 Department of Chemistry and Bioscience, Section of Biology and Environmental Science, Aalborg University, Fredrik Bajers Vej 7H, DK-9220 Aalborg E, Denmark; 2 Department of Bioscience, Section of Genetics, Ecology and Evolution, Aarhus University, Ny Munkegade 114, DK-8000 Aarhus C, Denmark

## Abstract

Multiple environmental factors acting in concert can interact and strongly influence population fitness and ecosystem composition. Studies investigating interactions usually involve only two environmental factors; most frequently a chemical and another abiotic factor such as a stressful temperature. Here we investigate the effects of three environmental factors: temperature, an insecticide (dimethoate) and interspecific co-occurrence. We expose two naturally co-occurring species of *Drosophila (D. hydei* and *D. melanogaster*) to the different environments during development and examine the consequences on several performance measures. Results are highly species and trait specific with evidence of two- and three-way interactions in approximately 30% of all cases, suggesting that additive effects of combined environmental factors are most common, and that interactions are not universal. To provide more informative descriptions of complex interactions we implemented re-conceptualised definitions of synergism and antagonism. We found approximately equal proportions of synergistic and antagonistic interactions in both species, however the effects of interactions on performance differed between the two. Furthermore, we found negative impacts on performance in only 60% of interactions, thus our study also reveals a high proportion of cases with positive effects of interactions.

Natural populations are exposed to multiple environmental stimuli simultaneously^1^,^2^. The impact of environmental factors may vary, and this is especially pronounced in seasonally fluctuating environments, e.g. during winter^3^. Environmental factors may interact in their impact on organisms resulting in fitness consequences that are different from what would be expected when considering each factor individually^1^.

Interactions between environmental factors, both within and between biotic and abiotic factors, play an important role in determining species composition of communities and ecosystems^4^,^5^. Indeed, such interactions can be more important than abiotic habitat requirements when predicting community assemblies^6^, highlighting the importance of integrating interactions in ecological prediction models. Environmental factors may also interact with the genotype of individuals and the genetic constitution of populations. For instance, fitness consequences of inbreeding is typically exacerbated under stressful environmental conditions^7^, with proposed large implications for small and fragmented populations suffering from inbreeding and genetic drift^7^,^8^,^9^. Neglecting fitness consequences of interactions within and between biotic and abiotic interactions can have considerable undesirable consequences. This may result in underestimating the effect of multiple environmental factors on population persistency and the stability of communities^4^, a risk exemplified by the combination of thermal extremes and drought stress resulting from climate change^10^. The predictability and generalizability of responses to multiple environmental factors should be incorporated in general global climate change models^11^, and in ecological risk assessments^2^,^12^ for increased accuracy and prediction power of community assembly modelling^6^.

When the combination of deleterious environmental factors is more harmful to the organism than the sum of individual factors, this has traditionally been referred to as a “synergistic” interaction, e.g. higher temperature exacerbating the harmful effect of a pesticide^13^,^14^. If the combination of two or more factors is less harmful than the sum of individual factors, the interaction is traditionally termed “antagonistic”, e.g. insect herbivory reducing harmful effects of plant competition^15^. However, the validity of these relatively simple terms has been debated^1^,^16^,^17^, and several authors have proposed a reconceptualization of the typical classifications, enabling an inclusion of, e.g., interactions between individual environmental factors with opposing directions^18^,^19^.

Studies of interactions typically investigate only two environmental factors, and among these the most frequently investigated factors are different temperatures and presence or absence of a chemical^20^. Among more than 150 studies investigating two-way interactions between a chemical and another environmental factor, 74% find an interaction, with 93% of these interactions being synergistic and 7% antagonistic^2^. Interactions have also been identified when assessing fitness consequences of chemicals in combination with biotic stressors, e.g. interspecific competition^21^, starvation^22^, pathogens/parasites^23^ and predation^24^. In general, studies performed so far are highly biased towards assessing pesticides or other chemical compounds and typically involve only one species or several species investigated separately^2^,^25^,^26^.

In this study, we investigate consequences of exposure to biotic and abiotic factors, that are potentially stressful in isolation or in combination, in two cosmopolitan *Drosophila* species; *Drosophila hydei* and *Drosophila melanogaster*. The effects of three environmental factors were investigated; low, intermediate and high developmental temperatures, presence or absence of the organophosphate insecticide dimethoate, and presence or absence of a co-occurring species. We combined all environmental factors in a full factorial manner, and analysed potential synergistic and antagonistic interactions (Fig. 1). The effects of the three environmental factors were investigated by assessing egg-to-adult viability, developmental time, upper and lower thermal limits and a behavioural trait. A composite measure of performance across traits was also computed. Responses to unfavourable environmental conditions can be highly sex-specific^27^,^28^ and therefore both females and males were assayed. Based on recent reviews suggesting a high frequency of interactions, as well as the seemingly prevalent notion, that synergistic interactions typically have negative impacts on performance, we hypothesised to find 1) multiple environmental factors primarily interact in their effect on performance, 2) interactions are primarily synergistic, 3) the effects of interactions are mostly negative, and 4) the frequency and direction of interactions are both trait and species specific.

## Results

Given the large amount of data and high number of potential interactions, we aimed at quantifying general patterns of responses to individual and combined environmental factors. We will refer to the environment experienced by the flies as the treatment, which is thus composed of up to three manipulated environmental factors. The consequences of the different treatments on individual traits as well as on the composite performance are summarized in Table 1. In order to achieve an overall picture of interactions we constructed linear models and extracted the standardised model coefficients of all treatments on the different traits, which are summarized for *D. hydei* in Fig. 2 and for *D. melanogaster* in Fig. 3.

Across all traits we observed both benefits and costs of exposure to potentially stressful environments (Table 1). We observed large variation in the composite performance measure both within and across the treatments (Table 1). In *D. hydei*, co-occurrence with *D. melanogaster* caused a significantly improved performance in all traits except for Critical Thermal minimum (CT_min_). Conversely, *D. melanogaster* was largely unaffected by the presence of *D. hydei*. Dimethoate affected negative geotaxis behaviour as the sole trait in *D. melanogaster*, while in *D. hydei*, the presence of dimethoate significantly affected egg-to-adult viability, developmental time, and negative geotaxis behaviour. Egg-to-adult viability of *D. melanogaster* was largely unaffected by developmental temperature, whereas in *D. hydei* this trait was greatly impacted by heat alone, and also by cold when combined with other environmental factors (Table 1). In terms of the effect of developmental temperature on thermal tolerance, we also found different results in the two species. While a low developmental temperature resulted in a high cold tolerance and low heat tolerance in *D. melanogaster*, exposure of *D. hydei* to low developmental temperature resulted in significantly higher cold tolerance and unaltered heat tolerance compared to flies developed at an intermediate temperature, confirming results from other studies providing evidence for thermal acclimation^29^. The two species responded similarly in cold and heat tolerance to development at a high temperature, i.e. in both species high developmental temperature resulted in decreased CT_min_ but increased CT_max_. Overall, the consequences of the different factors were both species and trait specific, and in a few cases sex specific (Table 1).

In *D. hydei* 37% of all tests resulted in significant two- or three-way interactions (Table 2). In *D. melanogaster* we found significant interactions in only 19% of the cases. These interactions were almost equally distributed between synergistic and antagonistic interactions in both species. The proportion of positive and negative synergistic interactions was approximately equally frequent in both species, whereas the pattern of antagonistic interactions differed more between the two species. The majority of antagonistic interactions for *D. hydei* were positive antagonistic (80%), i.e. less positive than expected additively, whereas negative antagonistic interactions were the most frequent type of antagonism in *D. melanogaster* (80%). This resulted in differences in the effects of interactions on performance. For *D. hydei*, most interactions had a negative effect (72%), while in *D. melanogaster* the majority of interactions had a positive effect (67%). Overall, we found more interactions that affected performance negatively. When we observed a significant interaction in both sexes it was always of the same interaction type, however we also found some interactions that only affected one sex (Figs 2 and 3). In *D. melanogaster* in particular, the effects of interactions on developmental time and negative geotaxis seemed to differ between sexes (Fig. 3).

For all traits where the response to co-occurrence was significantly different from that of the control (25 °C, no co-occurrence and no dimethoate), co-occurrence was beneficial to *D. hydei,* whereas *D. melanogaster* was largely unaffected by co-occurrence (Table 1). Interestingly, in terms of composite performance, combinations of factors involving co-occurrence were mostly positive in both species, albeit highly variable between treatments. The effect of co-occurrence was typically dependent on other factors, e.g. for egg-to-adult viability of *D. hydei* co-occurrence of *D. melanogaster* increased the proportion of surviving adults at both 25 and 13 °C, but not at 31 °C (Fig. 4). However, at both 25 and 13 °C the addition of dimethoate resulted in lower egg-to-adult survival than expected from the effects of dimethoate alone at these temperatures, i.e. there were strong negative synergistic interactions (S−; Fig. 2). Heat (31 °C) reduced egg-to-adult viability but neither co-occurrence or dimethoate alone had a strong effect on survival at this temperature, however, when combined the resulting survival was comparable to that of the control treatment (Fig. 4). The interaction between heat, co-occurrence and dimethoate in *D. hydei* was classified as a negative antagonistic interaction (A−) as it was less negative than predicted additively, i.e. the interaction itself was beneficial in terms of survival (Fig. 2).

Dimethoate decreased the egg-to-adult survival of *D. hydei,* but was beneficial in terms of shortened developmental time and increased negative geotaxis behaviour. The egg-to-adult survival of *D. melanogaster* was not significantly decreased, and most other traits were also unaffected by the presence of dimethoate. Interestingly, in *D. melanogaster* the two sexes responded quite differently to dimethoate in their negative geotaxis behaviour response. Males showed an increased activity in the geotaxis assay, whereas females had a lower activity compared to the control treatment (Table 1). Lastly, the consequences of heat and cold were highly species and trait specific. For instance, *D. melanogaster* responded negatively to cold in all traits except egg-to-adult viability and CT_min_. Developmental temperature seemed to have the greatest impact on developmental time, CT_min_, and CT_max_ responses regardless of the co-occurrence and dimethoate status of the treatment (Table 1).

## Discussion

The main aim of this study was to quantify the frequency, magnitude and direction of interactions between a set of environmental factors. This was investigated by exposing *D. hydei* and *D. melanogaster* to different developmental temperatures, the insecticide dimethoate and co-occurrence of the two species. The effects of these environmental factors were investigated on a range of traits in a full factorial manner.

We found that the effects of individual environmental factors as well as effects of the combinations were highly species specific (Figs 2 and 3). This is in agreement with other studies finding species specific effects of multiple stressors^30^,^31^, suggesting that the exact effects of interactions depend on the experimental setup, as well as on the species investigated. This highlights the potential problems of extrapolating results from studies investigating interactions on one species to, e.g., community scale or to ecological risk assessments^12^, and thereby emphasizing the need for further studies using standardised methods and/or multiple species. Interestingly, we found that the effects of the different treatments on composite performance were largely similar in the two species (Table 1), albeit highly variable between treatments. The calculation of a composite overall performance measure allows for an unbiased comparison of treatments in relation to fitness^32^,^33^. However, care must be taken when interpreting such a measure as a component of fitness as it can be questioned whether some traits contribute more to fitness than others (in our calculations all traits were given the same weight).

**Figure 1:**
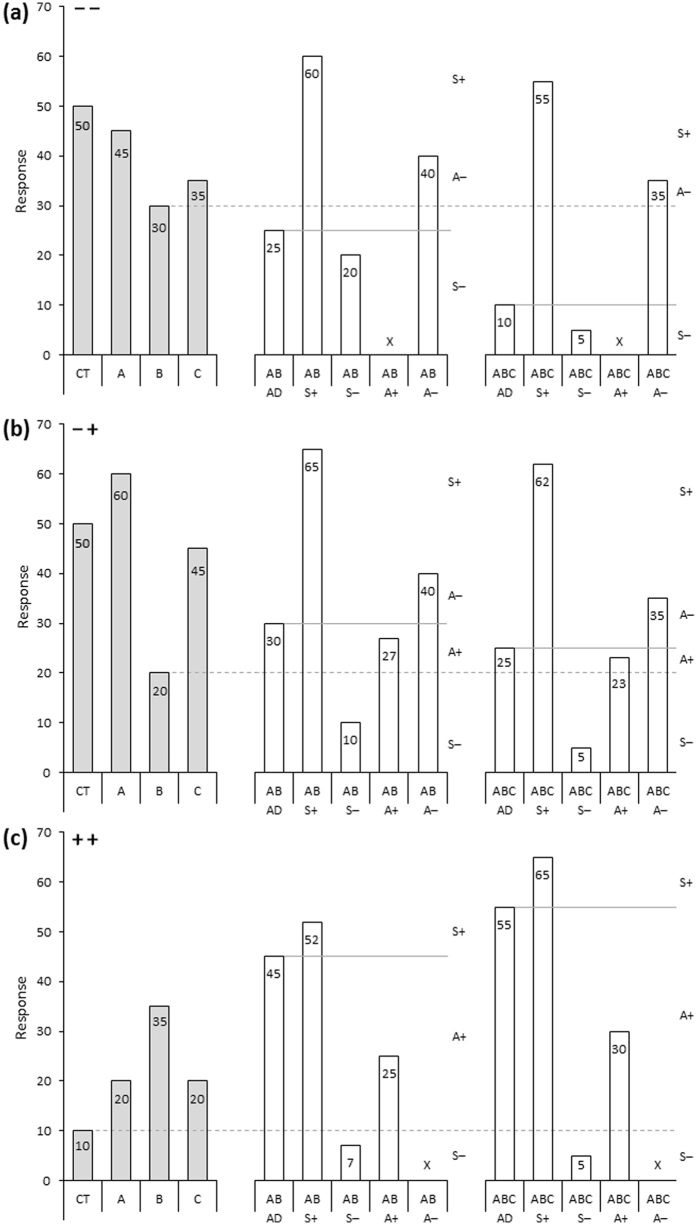
Illustration of our conceptual definitions of interaction types. Our definitions combine the magnitude and response direction of the interaction effect. Treatments in the factorial design include control (CT), with individual factors A, B, and C and with two factors (AB) or three factors (ABC). Directional interaction classes are +Synergistic (S+), −Synergistic (S−), +Antagonistic (A+), and −Antagonistic (A−) which depend on the effect of multiple factors (AB or ABC) compared to the additive sum (AD) of the individual effects of A and B (and C) relative to the control (CT). Height of the bars represents the absolute value of the response to each treatment. Grey shaded bars represent control treatment and the individual factors A, B, and C. Solid horizontal lines illustrate the additive sum for reference. The dashed horizontal lines represent the individual factor with the lowest response. The three plots illustrate interactions types in situations where the effects of individual environmental factors are all negative (**a**), opposing (**b**), and all positive (**c**) on the trait in question. An X indicates that the interaction class is not applicable in a given situation. Redrawn from refs 18, 19.

**Figure 2:**
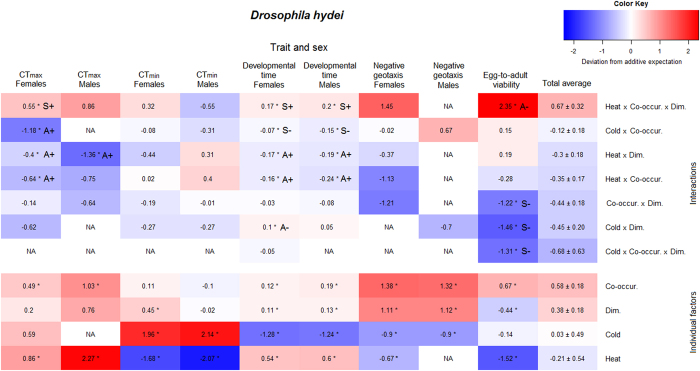
Heat map of interactions in *D. hydei*. Heat map showing the direction and magnitude of the model coefficients reflecting the effects of treatments on egg-to-adult viability, developmental time, CT_min_, CT_max_ and negative geotaxis (RING assay) in *D. hydei*. The effects are shown for both sexes in all traits except egg-to-adult viability. Positive coefficients represent positive deviation from the additive expectation, and can thus be interpreted as a performance advantage of the interaction itself, regardless of whether the treatment overall was beneficial in terms of performance when compared to the control. Contrary, a negative coefficient implies a negative deviation from additivity and that the interaction itself is detrimental to performance for a given trait. The direction of the effect is illustrated by colour shading from blue (negative) to red (positive) and the values indicate the strength of the effects. The upper part includes all two- and three-way interactions between heat or cold, co-occurrence (Co-occur.), and dimethoate (Dim.). The lower part includes all effects of the individual factors. Within each part the treatments (rows) have been sorted by the average total effect, i.e. the average effect across traits ± S.E., in descending order. An asterisk indicates a significant interaction, or a significant effect of the individual environmental factor. S+ and S− designate interactions that are classified as positive or negative synergistic, respectively, as described in the text. A+ and A− designate interactions that are classified as positive or negative antagonistic, respectively. Some treatments did not yield enough live adult flies for assessing all traits or did not exceed the minimum number of flies accepted for assessing a trait. In a few traits the effect of an individual environmental factor could therefore not be determined, and the interactions involving the particular factor were omitted from the model. Both cases are designated NA.

**Figure 3:**
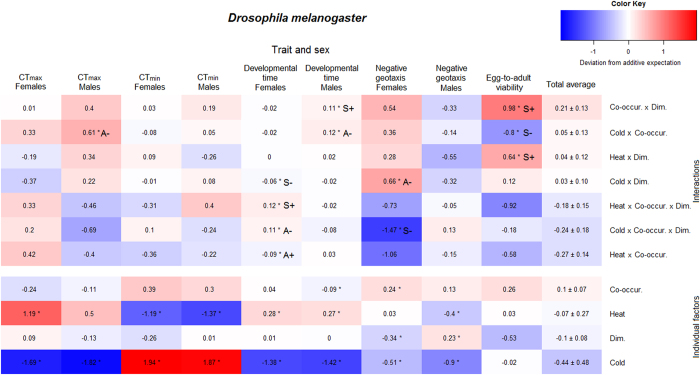
Heat map of interactions in *D. melanogaster.* Heat map showing the direction and magnitude of the model coefficients reflecting the effects of treatments on egg-to-adult viability, developmental time, CT_min_, CT_max_ and negative geotaxis (RING assay) in *D. melanogaster*. The effects are shown for both sexes in all traits except egg-to-adult viability. Positive coefficients represent positive deviation from the additive expectation, and can thus be interpreted as a performance advantage of the interaction itself, regardless of whether the treatment overall was beneficial in terms of performance when compared to the control. Contrary, a negative coefficient implies a negative deviation from additivity and that the interaction itself is detrimental to performance for a given trait. The direction of the effect is illustrated by colour shading from blue (negative) to red (positive) and the values indicate the strength of the effects. The upper part includes all two- and three-way interactions between heat or cold, co-occurrence (Co-occur.), and dimethoate (Dim.). The lower part includes all effects of the individual factors. Within each part the treatments (rows) have been sorted by the average total effect, i.e. the average effect across traits ± S.E., in descending order. An asterisk indicates a significant interaction, or a significant effect of the individual environmental factor. S+ and S− designate interactions that are classified as positive or negative synergistic, respectively, as described in the text. A+ and A− designate interactions that are classified as positive or negative antagonistic, respectively.

**Figure 4:**
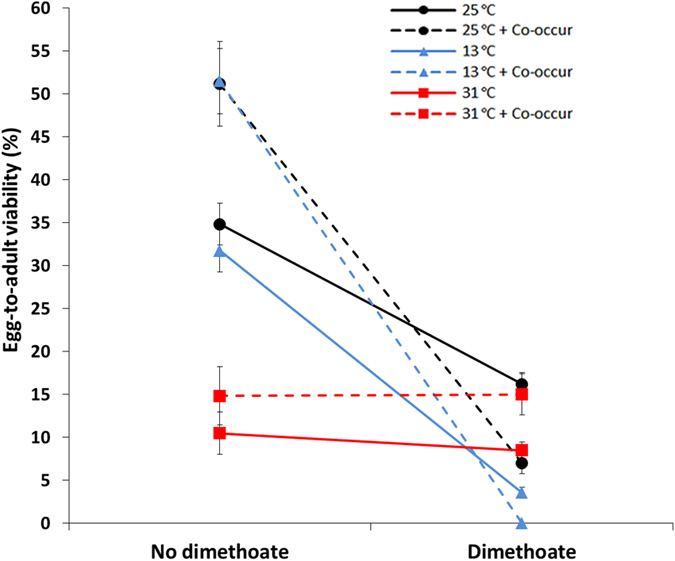
Example of combined effects of treatments. An example of the combined effects of temperature, co-occurrence (co-occur) and dimethoate on egg-to-adult viability (%) in *D. hydei*. Black lines represent 25 °C, blue are 13 °C, and red are 31 °C. Solid lines show the effects of temperature in the absence or presence of dimethoate (75 ppm). Dashed lines show the combined effects of temperature and co-occurrence in the absence or presence of dimethoate. Error bars represent standard error (S.E., *n* = 30–40).

In accordance with other studies our study provides evidence for the interaction of multiple environmental factors in their impact on fitness components^2^,^4^,^34^ (Figs 2 and 3 and Table 2). However, significant interactions were not the rule and in roughly 70% of all cases interactions were not observed, thus additive effects of multiple factors were the most frequent observation in this study. This is in contrast with the seemingly prevalent notion that interactions are more common than additive effects^2^,^18^,^34^,^35^. In the review by Holmstrup *et al*.^2^ of >150 studies investigating two-way interactions between a chemical and another environmental factor, 74% of the studies found interactions, and these were primarily synergistic. In a review by Crain *et al*.^18^ of >170 studies manipulating two or more environmental factors in coastal and marine ecosystems, interactions were similarly found in 74% of the studies, approximately equally distributed between synergistic and antagonistic interactions. Furthermore, there seems to be a bias towards investigating and reporting the interactive effects of two or more adverse individual environmental factors, i.e. of the all-negative interaction type (Fig. 1a), in ecological research and toxicology (e.g. reviewed in refs 34, 36 and 37). In general researchers tend to be biased towards publishing “positive” results, i.e. showing interactions rather than additive effects^2^, which could incorrectly reflect the frequency of interactions in nature and in laboratory studies.

The majority of studies investigating the effects of environmental factors and their potential interactions have used survival as a metric^34^,^35^. Less frequently, researchers have examined the effects of interactions on sub-lethal parameters such as growth, reproduction, behaviour or biochemical biomarkers, in which effects can potentially be detected before they affect survival^14^,^38^. We found that the effects of individual and combined environmental factors varied greatly depending on the trait being investigated (Figs 2 and 3), and consequently the overall performance measure was highly variable. In both species we found examples of significant effects of treatment on developmental time, thermal tolerances and behaviour without a notable effect on egg-to-adult survival. This context dependency raises concerns about drawing conclusions about the severity of interactions in studies that are based solely on mortality assays.

In our study, *D. hydei* benefitted strongly in almost all traits, including composite performance, from co-occurring with *D. melanogaster*, which to a lesser extent benefitted from developing with *D. hydei*. Studies on co-occurrence and interspecific competition in *Drosophila* are relatively scarce (but see e.g. refs 39, 40 and 41) and we have been unable to find studies investigating the interactions between co-occurrence or competition and other environmental variables in *Drosophila*. The egg-to-adult viability data for *D. hydei* suggests that the environmental conditions are suboptimal for full development in the control treatment (35% egg-to-adult survival), but when co-occurring with *D. melanogaster* (except at 31 °C) survival is significantly higher. We propose that *D. melanogaster* enhances the medium for the slower developing *D. hydei*, e.g. by increasing porosity or nutrient availability, thus resulting in positive effects of co-occurrence. These patterns were also evident in the composite performance measure, which was positive in the majority of cases, especially for *D. hydei*.

With the increasing realization of the importance of complex interactions in ecological contexts, it has become clear that despite the common use of the terms synergism and antagonism in the scientific literature to describe interactions, consensus seems to be lacking regarding an operational definition^1^,^2^,^16^,^17^,^18^,^19^,^35^,^42^. Synergism is normally used to define a cumulative effect greater than the additive sum of individual effects, whereas antagonism defines a cumulative effect that is less than additive^1^. Traditionally, the differentiation between synergism and antagonism has been relatively straightforward when individual factors are unidirectional, i.e. all-negative or all-positive (Fig. 1a–c 1,16), however problems arise when individual factors are of opposite directions (Fig. 1b).

Because of the challenges arising from 1) the typical direction-independent classifications, and 2) opposing individual effects, we employed an alternative approach that systematically defines synergism and antagonism based on the direction and magnitude of the cumulative effect (Fig. 1), as proposed by Piggott *et al*.^19^. To highlight the specific challenges faced in the traditional interaction framework, we attempted to re-designate interactions in our dataset by the classic definitions, and elaborated on situations where the challenge in using such terms can be circumvented by the re-conceptualised ideas (see Supplementary Figures S4 and S5 and accompanying discussion). In contrast to classic definitions, the proposed re-conceptualised model applies positive or negative to synergism or antagonism, representing situations where cumulative effects are more positive or more negative than additive (for synergistic interactions) or less positive or less negative than additive (for antagonistic interactions) providing additional information of the direction of interactions. We emphasize that the prefixes *positive* and *negative* do not describe the performance or fitness effect of the interaction, e.g. a *positive antagonism* is not necessarily beneficial to the organism, partly because it can be difficult to establish whether an effect direction is beneficial or detrimental to fitness in some traits, e.g. as in the case of developmental time^43^. We expanded on the original reconceptualization of these classic terms by applying them to three-way interactions (Fig. 1). Such second-order interactions of three or more factors have rarely been investigated (but see refs 11, 44 and 45) perhaps due to complicated experimental designs and complex interpretation of such interactions^35^. The new directional interaction type approach overcomes some of the problems of the traditional framework. We argue that despite looking complex at first, this approach translates into more informative descriptions and eases interpretation^18^,^19^.

In this study we found a large proportion of treatments that were beneficial relative to the control treatment. Often treatments had negative effects on some traits, but led to performance benefits in others, i.e. highly context dependent, complicating the traditional assumption of detrimental synergy in situations of opposing individual factors^18^. Our finding of positive effects of interactions on performance is in accordance with other studies showing that the frequency of interactions with a beneficial effect can increase with the amount of detrimental environmental factors and that positive rather than negative effects of interactions dominate in certain natural communities^46^,^47^. Moreover, positive interactions can maintain the diversity of harsh environments where mutualistic relationships between species often govern survival^48^,^49^. Thus, our study adds to the growing realization of the importance of positive interactions in ecology which should be taken into account in ecological models and predictions. Furthermore, we identified several situations where an environmental factor, when applied alone, had little or no effect on performance, but when combined with other environmental factors resulted in a significant effect on performance. In (eco)toxicology this is sometimes referred to as potentiation or sensitisation^50^. Such interactions are of great interest, especially in environmental risk assessments, because factors that are seemingly harmless can, when combined, have tremendous unpredictable effects^2^,^19^,^35^.

## Conclusion

In this study we investigated effects of three environmental factors (temperature, dimethoate and species co-occurrence) and their interactions on several life history traits, thermal resistance and a behavioural trait in both sexes of two *Drosophila* species. We expand on the scarce knowledge on consequences of interactions between more than two environmental factors. In doing so, we take novel steps to provide more informative descriptions of consequences of such complex interactions on fitness components. Results suggest that although interactions do occur they are not omnipresent and additivity is more often observed. Further extrapolating results from one species, trait or sex to others might yield misleading results. Lastly, our study also highlights the importance of considering positive interactions in ecological contexts.

## Methods

### Fly stocks and preparation

*D. hydei* and *D. melanogaster* mass bred populations were established from flies caught at an apple heap in Karensminde orchard at the Danish peninsula of Jutland in September 2014 and November 2013 respectively (for details on location and habitat see ref. 51). Wild caught inseminated females (n = 25) contributed with an equal number of offspring to the establishment of mass bred populations. Each population was maintained at a population size of minimum 1000 individuals per generation at 25 °C in a 12:12 light:dark photoperiod for 8 (*D. hydei*) and 37 (*D. melanogaster*) generations prior to the experiment. The medium used for maintenance of flies was a standard *Drosophila* medium consisting of yeast (16 g/L), soy flour (9 g/L), cornmeal (66 g/L), agar (5 g/L), and glucose syrup (100 g/L) mixed with tap water. To control fungal growth, nipagen (9 mL/L) and 80% acetic acid (1 mL/L) were added to the medium. Parental flies were density controlled during development by controlled egg-laying time (24 h period) of 200 flies in five 175 mL bottles with 35 mL medium. To density control the development of experimental flies, we collected eggs produced by parental flies (12–14 days old for *D. hydei* and 3–4 days old for *D. melanogaster*) on three consecutive days using the following approach: Twenty parental flies were distributed into each of 50 vials at 25 °C, each containing a spoon with 1.5 mL of *Drosophila* medium with dry yeast. Approximately 12 h later, eggs were transferred in groups of 40 to vials containing 9 mL Formula 4–24^®^ Instant *Drosophila* Medium Blue (Carolina Biological Supply Company, Burlington, NC, USA). This medium was mixed the day before egg collection and consisted of 1.6 g Formula 4–24^®^ instant medium and 7.5 mL demineralised water (with or without dimethoate), and was kept at 10 °C until use. Instant medium was used in all treatments, regardless of dimethoate status.

### Experimental setup

We investigated the effects of three different developmental environmental factors and the potential interactions between these factors on multiple traits, by exposing developing flies to different temperatures, an insecticide and co-occurrence in a full factorial design (Supplementary Table S1). Co-occurrence in this case does not necessarily imply competition between the two species and thus potentially fitness costs, so in this study we simply refer to this situation as co-occurrence. Flies were exposed to either of three constant temperatures during the development from egg to adult; 13 (cold), 25 (intermediate) and 31 °C (warm). This was done by transferring medium vials, immediately after egg collection, to climate incubators (Binder model KBWF 720 E5.3, Binder, Tuttlingen, Germany) maintaining an average (±s.d.) temperature of 13 ± 0.2, 25 ± 0.5 or 31 ± 0.5 °C, 40–60%RH and a 12:12 light:dark photoperiod under cool white-fluorescent light. *D. hydei* and *D. melanogaster* are cosmopolitan species that can be found consistently from latitudes −46.2 °S to +73.4 °N and −54.5 °S to +73.4 °N, respectively (TaxoDros database: v.1.04, http://www.taxodros.uzh.ch). Within these distributional ranges they can experience temperatures in the range of −8.4 °C to +33.5 °C and −9.0 °C to +33.6 °C, for *D. hydei* and *D. melanogaster*, respectively (WorldClim database: v.1.4, http://www.worldclim.org)^52^. Climatic temperature ranges were 10% quantile of minimum temperature in coldest month and 90% quantile of maximum temperature in warmest month. Data was treated and cross-referenced using methods described in Schou *et al*. (in press)^29^. Thus the thermal regimes employed in this study are well within the range of what the two species will experience in their natural habitats.

As a chemical abiotic environmental factor we used dimethoate (analytical grade 99.5%, CAS: 60-51-5, Sigma-Aldrich, Seelze, Germany; for more information on dimethoate e.g. rates of breakdown and acidity see Kristensen *et al*.^53^ and references therein). A 10 ng μL^−1^ stock solution of dimethoate was prepared in demineralised water the day before the experiments, and mixed with the developmental medium for a nominal concentration in the medium of 75 μg L^−1^ (ppb). This concentration was based on data from Kristensen *et al*.^53^ and a series of preliminary range-finding tests assessing egg-to-adult viability and developmental time. *D. hydei* and *D. melanogaster* showed dissimilar responses both in terms of viability and developmental time in preliminary experiments, and we therefore selected an intermediate concentration (Supplementary Figures S1 and S2). Although dimethoate has been banned by the European Union and the US Environmental Protection Agency^54^, it is still widely used, especially in developing countries and illegally in Southern Europe^54^,^55^, where concentrations of up to 120 ppb have been found in olives^55^. Thus, the concentration used in this study is considered ecologically relevant, i.e. it is within those concentrations encountered by insects in the field.

Co-occurrence during development was imposed by placing an equal number of eggs from *D. hydei* and *D. melanogaster* simultaneously in the vials (Supplementary Table S1). The total number of eggs was the same as in treatments without co-occurrence, reducing potential density-dependent effects during development. We refer to the 25 °C, no co-occurrence and no dimethoate treatment as the control treatment. For most treatments, we collected eggs into 30 vials per treatment, and for some treatments, which were expected to yield few surviving adult flies, we collected eggs into 40 vials per treatment (Supplementary Table S1). In total 24,000 eggs were distributed to 600 vials. The vials were placed randomly in racks and the racks within each incubator were shuffled randomly every day until emergence of adult flies. The combination of three temperatures (13, 25 and 31 °C), two dimethoate levels (0 and 75 μg L^−1^ (ppb)), and two co-occurrence levels (no co-occurrence and co-occurrence) resulted in a total of 12 treatments per species. The vials from each developmental rearing regime were checked daily at 08:00 a.m. for emerged flies. Emerged flies were anaesthetised with CO_2_, sexed and counted to estimate egg-to-adult viability and developmental time. Flies needed for phenotypic assessments were transferred in groups of 40 flies to separate vials for each sex and to a common environment two days prior to assessments. In the common environment, adult flies from the two species were kept separately at 7 mL standard *Drosophila* medium (same as used for maintenance) without dimethoate and at 25 °C.

### Phenotypes assessed

#### Egg-to-adult viability and developmental time

Egg-to-adult viability was determined as the proportion of eggs in a vial developing successfully into the adult life stage. Flies that had died during emergence from the pupae were not counted as a surviving adult fly. Developmental time was assessed every 24 h as the difference between time of emergence and time of egg collection. The assessment of developmental time of flies of a given treatment ceased when no flies had emerged for two consecutive days (four days for cold treatments). We defined a decreased egg-to-adult viability as a fitness disadvantage and interpreted a decreased developmental time (higher rate of development) as a fitness benefit. The ‘faster is better’ interpretation is debatable, as fast growth can be associated with trade-offs with other fitness components such as decreased efficiency of the immune system^43^, and costs and benefits associated with fast development is likely environment specific.

#### Thermal limits

Flies from each treatment were assessed for critical thermal minimum (CT_min_) and critical thermal maximum (CT_max_), i.e. their ability to tolerate low and high temperatures, respectively. These standardised procedures of gradual cooling or heating have been suggested to be more ecologically relevant than procedures using abrupt temperature changes^56^,^57^. We defined CT_min_ as the temperature at which absolutely no movement of the body or appendages of flies is observed (see e.g. ref. 56 for details), as a result of the flies entering chill coma. Similarly, CT_max_ or knockdown temperature is defined as the temperature at which a complete cessation of movement of the flies occurs, due to heating. From each sex and each treatment, 20 flies (for exact numbers see Supplementary Table S2) of age 60 ± 12 h were transferred to individual small glass vials (45 × 15 mm) and randomly placed in a metal rack, which was submerged in a water bath pre-set at 25 °C. When assessing CT_min_, the temperature in the water bath was decreased at a rate of 0.1 °C/min and the temperature, at which the flies was completely immobilised due to chill coma, was recorded. When assessing CT_max_ the temperature in the water bath was increased at a rate of 0.1 °C/min and temperature at which absolutely no movement was observed was recorded. Movement was stimulated by shining a flashlight and gently prodding the vials with a metal rod. We defined an increased CT_max_ i.e. higher heat tolerance as beneficial to performance, as evident in other studies^57^,^58^. Similarly congruent with other studies, we defined decreased CT_min_ i.e. higher cold tolerance as advantageous in terms of performance, because there seems to be only few costs associated with higher cold tolerance^59^,^60^,^61^.

#### Negative geotaxis

In order to assess the effects of the different environmental factors on behaviour, we investigated the negative geotaxis behaviour of the flies. Negative geotaxis is an innate escape response where flies move in opposite direction of the force of gravity. The behaviour is typically elicited via mechanical stimulation by tapping the flies to the bottom of an empty vial and assessed as the velocity or distance moved by the flies when ascending the walls of the container. For this purpose, we utilized a modified version of the high-throughput Rapid Iterative Negative Geotaxis (RING) assay, developed by Gargano *et al*.^62^. In this assay digital photography is used to document negative geotaxis behaviour in multiple groups of flies simultaneously (for details on our version of the RING apparatus see Supplementary Figure S3). To assess negative geotaxis, 10 flies were transferred into each of 10 empty vials. Fresh new vials were used for each treatment as Nichols *et al*.^63^ found that flies in previously used vials will not climb to the same extent as in new vials. In total 20 flies of each sex from each treatment at age 60 ± 12 h were assessed in the RING assay. Ten minutes after the flies had been transferred to the vials all ten vials were loaded into the apparatus. One minute later the RING apparatus was forcefully knocked down three times in rapid succession to initiate the geotaxis response. A photo of the vertical position of the flies was captured exactly 3 s after eliciting the behaviour. Preliminary tests found the most differentiated response after 3 s, as after 5 s almost all flies had reached the top of the vials. This was performed a total of 5 times with 30 s pause in between. The vials were then rotated within the rack, and a trial of 5 images were captured at each position as described above, resulting in a total of 50 images of each vial. Images of the flies’ positions were captured with the camera of an iPhone 5 s with default camera timer options (8 Mp; Apple Inc., Cupertino, CA, USA). The camera was mounted 30 cm from the apparatus in all experiments. The median height of the flies within each vial was measured using ImageJ software (version 1.48; ref. 64). All RING experiments were conducted in a climate controlled room at 25 °C, 50%RH and at constant light. Negative geotaxis behaviour was assessed between 08:00 and 10:00 a.m. on each test day, as the locomotor activity in *Drosophila* exhibits a distinct circadian rhythm^65^. An increased negative geotaxis behaviour, i.e. the flies crawled higher, was interpreted as beneficial, because the ability to escape a potential stressful environment is of key importance for fitness^66^.

### Composite performance

As a combined measure of overall performance of the treatments we calculated a composite performance measure based on all the traits expressed as a single value. This was done by standardizing the response of the different treatments within each trait, thus expressing the response to a treatment in terms of standard deviations from the mean of all treatments within a specific trait (negative and positive deviations were assigned according to the interpretations of how each trait relates to performance). We then averaged these deviations of each treatment across traits. We did this separately for each sex except for viability where sex-differentiation is not possible. By doing this we give equal weight to all traits, and thus we obtain an unbiased estimate of overall performance, which we assume constitute a component of fitness. This use of a composite performance/fitness measure is similarly employed in other studies^32^,^33^.

### Statistical analysis

To estimate the two-way and three-way interactions including the cold treatment, we constructed a linear model (cold model) for each trait with the factorial fixed effects temperature (two levels: benign and cold), dimethoate (two levels: 0 and 75 ppm) and co-occurrence (two levels: presence/absence), as well as all two- and three-way interactions. To compare cold model interactions with interactions involving heat exposure, we constructed parallel models including the heat treatment instead of the cold exposure (heat model). Individual and interaction effects of dimethoate and co-occurrence were included in both models, and thus extracted from one of the two models. In all traits, the response variable was scaled to a z-distribution to ease comparability across traits. The purpose of these models was to obtain a standardized measure of single and interaction coefficients as well as corresponding 95% confidence intervals. Given the large amount of data and models as well as our aim of obtaining a quantification of general patterns, we found this to be an appropriate approach, as opposed to model reduction and p-value estimation. Significance of an interaction was defined as when the confidence interval did not overlap with 0. To ease the comparison of interactions among treatments and traits, we produced heat maps of the estimated coefficients using the R-package ‘gplots’^67^.

A positive coefficient represents a positive deviation from the additive expectation, and can thus be interpreted as a performance advantage of the interaction itself, regardless of whether the treatment overall was beneficial in terms of performance when compared to the control. Thus although the flies from a particular treatment may be performing worse than the control group flies, the positive interaction is a benefit, compared to the expected value without the interaction. Contrary, a negative coefficient implies that the interaction itself is detrimental to performance.

If no flies had emerged from a given treatment (e.g. “cold + dimethoate + co-occurrence”) the corresponding benign temperature treatment (“benign + dimethoate + co-occurrence”) was removed from the model to create a balanced model. In this case the model included only two two-way interactions: temperature*dimethoate and temperature*co-occurrence. For CT_min_ and CT_max_, data were analysed with a general linear model. Egg-to-adult viability data were analysed with a generalised linear model with a logit link function. We detected overdispersion in the model and corrected for this using a quasi-generalised linear model. Developmental time data were analysed with a generalised linear mixed effect model with a Poisson distribution. Replicate vials were included as a random effect, as flies from the same vial were not independent. RING data were analysed with a general linear mixed model with replicate vial, position of the vial in the rack and number of replicate picture (trial number) included as random effects. All statistical analyses were performed in R^68^ (v. 3.1.2), and mixed models were performed using the R-package ‘lme4’^69^. For a straightforward representation of the effects on performance of each individual treatment relative to the control, we used a simple pairwise comparison (Welch’s t-test). These results will serve as background information when interpreting the results of the analysis of interactions presented above. CT_max_ data were anti-log transformed to fulfil assumptions of parametric analysis.

### Classification of synergism and antagonism

In contrast to the traditional direction-independent framework^1^ we use a classification system based on that of Piggott *et al*.^19^, which combines the magnitude and response direction (+ or −) of interaction effects to define synergism and antagonism (Fig. 1). Our definition can be illustrated by assigning a positive effect of an individual effect as +1 and a negative effect as −1. We define a deviation from additive that is greater than the sum of individual environmental factors and greater than any individual effect in the same direction or an interaction effect greater than both individual effects in absolute terms as synergistic. We classify it as *positive synergistic* (S+) i.e. more positive than predicted additively when +1 + 1 > + 2 or −1 + 1 > 1 or −1 + − 1 > 0, or *negative synergistic* (S−) i.e. more negative than predicted additively when −1 + − 1 < − 2 or −1 + 1 < − 1 or +1 + 1 < 0. If an interaction deviates from additivity and is less than the sum of individual factors or less-than-or-equal-to any individual effect in the same direction we define the interaction as antagonistic. We classify it as *positive antagonistic* (A+) i.e. less positive than predicted additively when +1 + 1 is between 0 and 2 or −1 + 1 is between −1 and 0 (or equal −1), or *negative antagonistic* (A−) i.e. less negative than predicted additively when −1 + − 1 is between −2 and 0 or −1 + 1 is between 0 and 1 (or equal 1). The terms ‘more or less positive’ and ‘more or less negative’ also apply to situations where one individual environmental factor has no effect, and the definitions are also easily applied to three-way interactions (Fig. 1). With this definition synergistic and antagonistic does not relate to whether or not the interaction itself constitutes a performance benefit. To assess this, we determine whether the deviation from additivity is positive (performance advantage) or negative (performance disadvantage) based on the model coefficients as described above. In any case a significant interaction can be directly interpreted as an ecological interaction between the individual environmental factors.

## Additional Information

**How to cite this article**: Ørsted, M. *et al*. Biotic and abiotic factors investigated in two *Drosophila* species – evidence of both negative and positive effects of interactions on performance. *Sci. Rep.* **7**, 40132; doi: 10.1038/srep40132 (2017).

**Publisher's note:** Springer Nature remains neutral with regard to jurisdictional claims in published maps and institutional affiliations.

## Supplementary Material

Supplementary Information

## Figures and Tables

**Table 1:** Results for the effects of each treatment on egg-to-adult viability, developmental time, CT_min_, CT_max_, negative geotaxis (RING assay) and overall composite performance in *D. hydei* and *D. melanogaster.*

	Treatment	Egg-to-adult viability (%)	Developmental time (days)		CT_min_ (°C)		CT_max_ (°C)		Negative geotaxis (cm)		Composite performance	
		M/F	M	F	M	F	M	F	M	F	M	F
*D. hydei*	Control (25 °C)	34.8 ± 2.5	23.4 ± 0.19	22.4 ± 0.23	4.47 ± 0.08	4.85 ± 0.10	39.0 ± 0.13	39.0 ± 0.09	2.35 ± 0.09	2.4 ± 0.13	−0.11 ± 0.27	−0.10 ± 0.26
	Co-occur.	51.2 ± 5.0**	19.4 ± 0.23**	19.9 ± 0.23**	4.56 ± 0.10	4.76 ± 0.09	39.4 ± 0.05**	39.5 ± 0.03**	4.63 ± 0.08**	4.5 ± 0.10**	0.50 ± 0.24	**0.57 ± 0.24**
	Dim.	25.6 ± 1.4**	20.6 ± 0.21**	20.1 ± 0.22**	4.49 ± 0.07	4.46 ± 0.07**	39.3 ± 0.06	39.2 ± 0.06	4.35 ± 0.10**	3.8 ± 0.09**	0.20 ± 0.18	**0.16 ± 0.19**
	Co-occur. + Dim.	16.6 ± 2.5**	18.6 ± 0.20**	18.4 ± 0.21**	4.59 ± 0.08	4.53 ± 0.07**	39.4 ± 0.04*	39.6 ± 0.04**	4.37 ± 0.14**	4.3 ± 0.11**	0.20 ± 0.18	**0.31 ± 0.20**
	Cold	31.8 ± 2.5	80.9 ± 0.54**	80.0 ± 0.51**	1.82 ± 0.12**	2.48 ± 0.18**	39.5 ± 0.04**	**39.2 ± 0.14**	0.75 ± 0.05**	0.8 ± 0.06**	−0.10 ± 0.55	**−0.29 ± 0.49**
	Cold + Co-occur.	51.5 ± 3.9**	78.2 ± 0.72**	77.0 ± 0.77**	2.30 ± 0.13**	2.49 ± 0.15**	**39.2 ± 0.10**	**39.3 ± 0.12**	4.30 ± 0.11**	NA	0.29 ± 0.48	**0.23 ± 0.53**
	Cold + Dim.	6.5 ± 0.9**	68.1 ± 0.60**	65.6 ± 0.64**	2.17 ± 0.15**	2.42 ± 0.17**	**39.1 ± 0.10**	**39.2 ± 0.15**	1.49 ± 0.07**	NA	−0.42 ± 0.44	−0.22 ± 0.45
	Cold + Co-occur. + Dim.^a^	1.3 ± 0.4**	NA	67.7 ± 1.73**	NA	NA	NA	NA	NA	NA	0.95 ± 0.00	−0.99±0.02
	Heat	10.5 ± 2.5**	12.8 ± 0.14**	13.0 ± 0.13**	6.38 ± 0.18**	6.29 ± 0.12**	39.8 ± 0.09**	39.9 ± 0.08**	NA	1.3 ± 0.11**	0.01 ± 0.43	−0.19 ± 0.41
	Heat + Co-occur.	14.8 ± 3.4**	13.5 ± 0.22**	13.6 ± 0.19**	6.10 ± 0.09**	6.18 ± 0.14**	39.9 ± 0.07**	39.8 ± 0.11**	NA	NA	0.17 ± 0.42	−0.02 ± 0.36
	Heat + Dim.	8.3 ± 1.3**	13.7 ± 0.17**	13.8 ± 0.19**	6.11 ± 0.18**	6.28 ± 0.31**	39.6 ± 0.14**	39.7 ± 0.09**	2.74 ± 0.14*	NA	−0.14 ± 0.32	−0.14 ± 0.37
	Heat + Co-occur. + Dim.	29.5 ± 4.4	12.8 ± 0.15**	12.6 ± 0.17**	6.35 ± 0.11**	6.06 ± 0.09**	39.8 ± 0.05**	40.0 ± 0.07**	3.37 ± 0.09**	3.2 ± 0.07**	0.18 ± 0.35	**0.28 ± 0.34**
*D. melanogaster*	Control (25 °C)	80.0 ± 2.5	12.4 ± 0.10	12.3 ± 0.10	6.6 ± 0.07	6.6 ± 0.08	39.9 ± 0.08	40.1 ± 0.04	5.48 ± 0.10	4.0 ± 0.10	0.23 ± 0.17	**0.13 ± 0.15**
	Co-occur.	83.8 ± 2.8	13.8 ± 0.28**	11.8 ± 0.11**	6.4 ± 0.07	6.4 ± 0.10	39.9 ± 0.05	40.0 ± 0.06	5.62 ± 0.10	4.3 ± 0.08*	0.30 ± 0.15	**0.23 ± 0.15**
	Dim.	70.3 ± 4.8	12.4 ± 0.10	12.1 ± 0.10*	6.6 ± 0.11	6.7 ± 0.07	39.9 ± 0.06	40.1 ± 0.04	5.73 ± 0.08*	3.6 ± 0.11**	0.13 ± 0.23	−0.07 ± 0.20
	Co-occur. + Dim.	89.1 ± 1.5**	12.2 ± 0.08*	12.0 ± 0.08**	6.3 ± 0.07**	6.5 ± 0.08	40.0 ± 0.04	40.0 ± 0.05	5.51 ± 0.09	4.6 ± 0.10**	0.42 ± 0.16	**0.34 ± 0.19**
	Cold	79.7 ± 1.9	51.3 ± 0.28**	48.8 ± 0.23**	4.1 ± 0.11**	3.7 ± 0.08**	39.1 ± 0.04**	39.4 ± 0.09**	4.31 ± 0.11**	3.5 ± 0.09**	−0.33 ± 0.43	−0.26 ± 0.42
	Cold + Co-occur.	69.5 ± 3.3*	50.1 ± 0.37**	48.3 ± 0.40**	3.9 ± 0.14**	3.6 ± 0.09**	39.3 ± 0.07**	39.3 ± 0.09**	4.27 ± 0.16**	4.2 ± 0.09	−0.34 ± 0.42	−0.26 ± 0.45
	Cold + Dim.	72.3 ± 1.9*	52.6 ± 0.29**	51.3 ± 0.30**	4.0 ± 0.12**	3.9 ± 0.09**	39.1 ± 0.08**	39.2 ± 0.06**	4.14 ± 0.13**	3.8 ± 0.07**	−0.44 ± 0.43	−0.39 ± 0.44
	Cold + Co-occur. + Dim.	77.3 ± 2.5	49.1 ± 0.23**	46.3 ± 0.20**	4.0 ± 0.10**	3.6 ± 0.09**	39.2 ± 0.07**	39.3 ± 0.11**	3.91 ± 0.13**	3.6 ± 0.09**	−0.34 ± 0.42	−0.27 ± 0.43
	Heat	80.5 ± 2.1	9.5 ± 0.06**	9.3 ± 0.06**	7.3 ± 0.06**	7.2 ± 0.11**	40.2 ± 0.13	40.5 ± 0.06**	5.04 ± 0.08**	4.1 ± 0.09	**0.18 ± 0.28**	**0.23 ± 0.28**
	Heat + Co-occur.	75.0 ± 2.4	10.1 ± 0.11**	9.8 ± 0.10**	7.3 ± 0.08**	7.2 ± 0.07**	39.9 ± 0.13	40.6 ± 0.06**	5.02 ± 0.10**	3.1 ± 0.14**	0.02 ± 0.25	−0.05 ± 0.33
	Heat + Dim.	82.3 ± 1.2	9.4 ± 0.10**	9.2 ± 0.10**	7.5 ± 0.06**	7.3 ± 0.05**	40.2 ± 0.11	40.5 ± 0.07**	4.70 ± 0.12**	4.0 ± 0.08	**0.16 ± 0.32**	**0.22 ± 0.28**
	Heat + Co-occur. + Dim.	78.3 ± 2.4	9.1 ± 0.09**	8.8 ± 0.08**	7.1 ± 0.07**	7.5 ± 0.11**	**40.0 ± 0.14**	40.6 ± 0.05**	4.27 ± 0.10**	2.8 ± 0.11**	**0.003 ± 0.26**	−0.01 ± 0.38

Dim.: dimethoate. Co-occur.: co-occurrence. Values are expressed as means ± S.E. for males (M) and females (F). The direction of the effect of a given treatment on a trait in relation to performance compared to the control environment (25 °C, no dimethoate, no co-occurrence) for that trait is indicated; bold numbers indicate a performance advantage, numbers not in bold a disadvantage. Asterisks indicate significant difference from control: *p < 0.05, **p < 0.01 (Welch’s t-test). Note that these pairwise comparisons only reflect the effects on performance of the treatment, not the interaction between the two or three involved factors. Some treatments did not yield enough live adult flies for assessing all traits or did not exceed the minimum number of flies accepted for assessing a trait. These are indicated as NA. The number of flies (*n*) that each value is based on, as well as the minimum number of flies accepted for a given trait, can be found in Supplementary Table S2. The composite performance is calculated as the average effect of each treatment after standardising the responses within each trait, and thus represents the average performance effect of the different treatments across the five traits (±S.E., *n* = 5) for each sex. Thus, the direction of the effect of composite performance is not relative to the control. Bold numbers here represent a positive composite performance measure, and numbers that are not in bold represent a negative composite performance measure. Egg-to-adult viability is included in the estimate of composite performance of both males and females as sex-differentiation was not performed for this trait.

^a^This treatment yielded only few surviving females and not enough flies to assess thermal tolerance or negative geotaxis.

**Table 2:** Number of significant interactions in all combinations of trait and sex for all treatments in both species showing the number of two- and three-way interactions, the classification of the interactions and the fitness effect of the interactions.

		*D. hydei*	*D. melanogaster*	Total
Number of interactions	Total	18 (37)	12 (19)	30 (27)
	Two-way	13 (72)	9 (75)	22 (73)
	Three-way	5 (28)	3 (25)	8 (27)
Interaction classification	Synergistic	8 (44)	7 (58)	15 (50)
	S+	3 (16)	4 (33)	7 (23)
	S−	5 (28)	3 (25)	8 (27)
	Antagonistic	10 (56)	5 (42)	15 (50)
	A+	8 (45)	1 (9)	9 (30)
	A−	2 (11)	4 (33)	6 (20)
Performance effect of interaction	Positive	5 (28)	8 (67)	13 (43)
	Negative	13 (72)	4 (33)	17 (57)

Classification of interactions into positive and negative synergism (S+ and S−, respectively) and positive and negative antagonism (A+ and A−, respectively) is described in the text. A positive effect of an interaction on performance is defined as when the interaction is more positive than predicted additively and thus beneficial. Similarly, an interaction is defined as having a negative effect on performance when the interaction is more negative than predicted additively and therefore detrimental. The percentage of total interactions is given in parentheses after each number. For the ‘total’ row the number in parentheses designates the proportion of significant interactions among all tested potential interactions.

## References

[b1] Folt C., Chen C., Moore M. & Burnaford J. Synergism and antagonism among multiple stressors. Limnol. Oceanogr. 44, 864–877 (1999).

[b2] Holmstrup M. . Interactions between effects of environmental chemicals and natural stressors: a review. Sci. Total Environ. 408, 3746–3762 (2010). 19922980 10.1016/j.scitotenv.2009.10.067

[b3] Williams C. M., Henry H. A. L. & Sinclair B. J. Cold truths: how winter drives responses of terrestrial organisms to climate change. Biol. Rev. 90, 214–235 (2014). 24720862 10.1111/brv.12105

[b4] Relyea R. & Hoverman J. Assessing the ecology in ecotoxicology: a review and synthesis in freshwater systems. Ecol. Lett. 9, 1157–1171 (2006). 16972879 10.1111/j.1461-0248.2006.00966.x

[b5] Kéfi S., Holmgren M. & Scheffer M. When can positive interactions cause alternative stable states in ecosystems? Funct. Ecol. 30, 88–97 (2016).

[b6] Schuwirth N., Dietzel A. & Reichert P. The importance of biotic interactions for the prediction of macroinvertebrate communities under multiple stressors. Funct. Ecol. 30, 974–984 (2015).

[b7] Fox C. W. & Reed D. H. Inbreeding depression increases with environmental stress: an experimental study and meta-analysis. Evolution. 65, 246–258 (2011). 20731715 10.1111/j.1558-5646.2010.01108.x

[b8] Liao W. & Reed D. H. Inbreeding–environment interactions increase extinction risk. Anim. Conserv. 12, 54–61 (2009).

[b9] Schou M. F., Loeschcke V. & Kristensen T. N. Inbreeding depression across a nutritional stress continuum. Heredity 115, 56–62 (2015). 26059969 10.1038/hdy.2015.16 PMC4815500

[b10] Bellard C., Bertelsmeier C., Leadley P., Thuiller W. & Courchamp F. Impacts of climate change on the future of biodiversity. Ecol. Lett. 15, 365–377 (2012). 22257223 10.1111/j.1461-0248.2011.01736.x PMC3880584

[b11] Kaunisto S., Ferguson L. V. & Sinclair B. J. Can we predict the effects of multiple stressors on insects in a changing climate? Curr. Opin. Insect Sci. 17, 55–61 (2016). 27720074 10.1016/j.cois.2016.07.001

[b12] Bednarska A. J., Jevtić D. M. & Laskowski R. More ecological ERA: incorporating natural environmental factors and animal behavior. Integr. Environ. Assess. Manag. 9, e39–46 (2013). 23625590 10.1002/ieam.1444

[b13] Osterauer R. & Köhler H. R. Temperature-dependent effects of the pesticides thiacloprid and diazinon on the embryonic development of zebrafish (*Danio rerio*). Aquat. Toxicol. 86, 485–494 (2008). 18281107 10.1016/j.aquatox.2007.12.013

[b14] Ørsted M. & Roslev P. A fluorescence based hydrolytic enzyme activity assay for quantifying toxic effects of Roundup^®^ to *Daphnia magna*. Environ. Toxicol. Chem. 34, 1841–1850 (2015). 25809520 10.1002/etc.2997

[b15] Haag J. J., Coupe M. D. & Cahill J. F. Antagonistic interactions between competition and insect herbivory on plant growth. J. Ecol. 92, 156–167 (2004).

[b16] Dunne R. P. Synergy or antagonism-interactions between stressors on coral reefs. Coral Reefs 29, 145–152 (2010).

[b17] Vanhoudt N., Vandenhove H., Real A., Bradshaw C. & Stark K. A review of multiple stressor studies that include ionising radiation. Environ. Pollut. 168, 177–192 (2012). 22634132 10.1016/j.envpol.2012.04.023

[b18] Crain C. M., Kroeker K. & Halpern B. S. Interactive and cumulative effects of multiple human stressors in marine systems. Ecol. Lett. 11, 1304–1315 (2008). 19046359 10.1111/j.1461-0248.2008.01253.x

[b19] Piggott J. J., Townsend C. R. & Matthaei C. D. Reconceptualizing synergism and antagonism among multiple stressors. Ecol. Evol. 5, 1538–1547 (2015). 25897392 10.1002/ece3.1465 PMC4395182

[b20] Heugens E. H. W., Hendriks A. J., Dekker T., Straalen N. M. van & Admiraal W. A review of the effects of multiple stressors on aquatic organisms and analysis of uncertainty factors for use in risk assessment. Crit. Rev. Toxicol. 31, 247–284 (2001). 11405441 10.1080/20014091111695

[b21] Boone M. D. & Semlitsch R. D. Interactions of an insecticide with competition and pond drying in amphibian communities. Ecol. Appl. 12, 307–316 (2002).

[b22] Spadaro D. A., Micevska T. & Simpson S. L. Effect of nutrition on toxicity of contaminants to the epibenthic amphipod *Melita plumulosa*. Arch. Environ. Contam. Toxicol. 55, 593–602 (2008). 18340476 10.1007/s00244-008-9153-2

[b23] Forson D. D. & Storfer A. Atrazine increases ranavirus susceptibility in the tiger salamander, Ambystoma tigrinum. Ecol. Appl. 16, 2325–2332 (2006). 17205907 10.1890/1051-0761(2006)016[2325:airsit]2.0.co;2

[b24] Relyea R. A. & Mills N. Predator-induced stress makes the pesticide carbaryl more deadly to gray treefrog tadpoles (*Hyla versicolor*). Proc. Natl. Acad. Sci. USA 98, 2491–2496 (2001). 11226266 10.1073/pnas.031076198 PMC30165

[b25] MacAlpine J. L. P., Marshall K. E. & Sinclair B. J. The effects of CO_2_ and chronic cold exposure on fecundity of female *Drosophila melanogaster*. J. Insect Physiol. 57, 35–37 (2011). 20868691 10.1016/j.jinsphys.2010.09.003

[b26] Rumschlag S. L., Boone M. D. & Fellers G. The effects of the amphibian chytrid fungus, insecticide exposure, and temperature on larval anuran development and survival. Environ. Toxicol. Chem. 33, 2545–2550 (2014). 25098758 10.1002/etc.2707

[b27] Hoffmann A. A., Hallas R., Anderson A. R. & Telonis-Scott M. Evidence for a robust sex-specific trade-off between cold resistance and starvation resistance in *Drosophila melanogaster*. J. Evol. Biol. 18, 804–810 (2005). 16033551 10.1111/j.1420-9101.2004.00871.x

[b28] Sørensen J. G., Kristensen T. N., Kristensen K. V. & Loeschcke V. Sex specific effects of heat induced hormesis in Hsf-deficient *Drosophila melanogaster*. Exp. Gerontol. 42, 1123–1129 (2007). 17950551 10.1016/j.exger.2007.09.001

[b29] Schou M. F., Mouridsen M. B., Sørensen J. G. & Loeschcke V. Linear reaction norms of thermal limits in *Drosophila*: predictable plasticity in cold but not in heat tolerance. Funct. Ecol. doi: 10.1111/1365-2435.12782 (2016).

[b30] Amarasekare K. G. & Edelson J. V. Effect of temperature on efficacy of insecticides to differential grasshopper (Orthoptera: Acrididae). J. Econ. Entomol. 97, 1595–1602 (2004). 15568348 10.1603/0022-0493-97.5.1595

[b31] Muturi E. J., Lampman R., Costanzo K. & Alto B. W. Effect of temperature and insecticide stress on life-history traits of *Culex restuans* and *Aedes albopictus* (Diptera: Culicidae). J. Med. Entomol. 48, 243–250 (2011). 21485359 10.1603/me10017

[b32] Overgaard J., Kearney M. R. & Hoffmann A. A. Sensitivity to thermal extremes in Australian *Drosophila* implies similar impacts of climate change on the distribution of widespread and tropical species. Global Change Biol. 20, 1738–1750 (2014). 10.1111/gcb.12521 24549716

[b33] Hereford J. A quantitative survey of local adaptation and fitness trade-offs. Am. Nat. 173, 579–588 (2009). 19272016 10.1086/597611

[b34] Darling E. S. & Côté I. M. Quantifying the evidence for ecological synergies. Ecol. Lett. 11, 1278–1286 (2008). 18785986 10.1111/j.1461-0248.2008.01243.x

[b35] Laskowski R. . Interactions between toxic chemicals and natural environmental factors - a meta-analysis and case studies. Sci. Total Environ. 408, 3763–3774 (2010). 20156639 10.1016/j.scitotenv.2010.01.043

[b36] Ban S. S., Graham N. A. J. & Connolly S. R. Evidence for multiple stressor interactions and effects on coral reefs. Glob. Chang. Biol. 20, 681–697 (2014). 24166756 10.1111/gcb.12453

[b37] Klaminder J., Jonsson M., Fick J., Sundelin A. & Brodin T. The conceptual imperfection of aquatic risk assessment tests: highlighting the need for tests designed to detect therapeutic effects of pharmaceutical contaminants. Environ. Res. Lett. 9, 84003 (2014).

[b38] Forbes V. E., Palmqvist A. & Bach L. The use and misuse of biomarkers in ecotoxicology. Environ. Toxicol. Chem. 25, 272–280 (2006). 16494252 10.1897/05-257r.1

[b39] Barker J. S. F. & Podger R. N. Interspecific competition between *Drosophila melanogaster* and *Drosophila simulans*: effects of larval density on viability, developmental period and adult body. Ecology 51, 170–189 (1970).

[b40] Grimaldi D. & Jaenike J. Competition in natural populations of mycophagous *Drosophila*. Ecology 65, 1113–1120 (1984).

[b41] Joshi A. Variation in the relative magnitude of intraspecific and interspecific competitive effects in novel versus familiar environments in two *Drosophila* species. J. Genet. 83, 179–188 (2004). 15536257 10.1007/BF02729895

[b42] Chou T. C. Drug combination studies and their synergy quantification using the Chou-Talalay method. Cancer Res. 70, 440–446 (2010). 20068163 10.1158/0008-5472.CAN-09-1947

[b43] Niemelä P. T., Vainikka A., Hedrick A. V. & Kortet R. Integrating behaviour with life history: boldness of the field cricket, *Gryllus integer*, during ontogeny. Funct. Ecol. 26, 450–456 (2012).

[b44] Chen C. Y., Hathaway K. M. & Folt C. L. Multiple stress effects of Vision herbicide, pH, and food on zooplankton and larval amphibian species from forest wetlands. Environ. Toxicol. Chem. 23, 823–831 (2004). 15095876 10.1897/03-108

[b45] Heugens E. H. W. . Population growth of *Daphnia magna* under multiple stress conditions: joint effects of temperature, food, and cadmium. Environ. Toxicol. Chem. 25, 1399–1407 (2006). 16704075 10.1897/05-294r.1

[b46] Callaway R. M. . Positive interactions among alpine plants increase with stress. Nature 417, 844–848 (2002). 12075350 10.1038/nature00812

[b47] Brooker R. W. . Facilitation in plant communities: The past, the present, and the future. Journal of Ecology 96, 18–34 (2008).

[b48] Cavieres L. A. & Badano E. I. Do facilitative interactions increase species richness at the entire community level? J. Ecol. 97, 1181–1191 (2009).

[b49] He Q., Bertness M. D. & Altieri A. H. Global shifts towards positive species interactions with increasing environmental stress. Ecol. Lett. 16, 695–706 (2013). 23363430 10.1111/ele.12080

[b50] Odum E. P. & Barrett G. W. Fundamentals of Ecology. (Thomson/Brooks/Cole, Belmont, CA, USA, 2005).

[b51] Schou M. F., Loeschcke V. & Kristensen T. N. Strong costs and benefits of winter acclimatization in *Drosophila melanogaster*. PLOS ONE 10, e0130307 (2015). 26075607 10.1371/journal.pone.0130307 PMC4468168

[b52] Hijmans R. J., Cameron S. E., Parra J. L., Jones P. G. & Jarvis A. Very high resolution interpolated climate surfaces for global land areas. Int. J. Climatol. 25, 1965–1978 (2005).

[b53] Kristensen T. N., Dahlgaard J. & Loeschcke V. Effects of inbreeding and environmental stress on fitness - using *Drosophila buzzatii* as a model organism. Conserv. Genet. 4, 453–465 (2003).

[b54] Galt R. E. Beyond the circle of poison: significant shifts in the global pesticide complex, 1976-2008. Glob. Environ. Chang. 18, 786–799 (2008).

[b55] Rastrelli L., Totaro K. & De Simone F. Determination of organophosphorus pesticide residues in Cilento (Campania, Italy) virgin olive oil by capillary gas chromatography. Food Chem. 79, 303–305 (2002).

[b56] Overgaard J., Kristensen T. N. & Sørensen J. G. Validity of thermal ramping assays used to assess thermal tolerance in arthropods. PLOS ONE 7, 1–7 (2012). 10.1371/journal.pone.0032758 PMC3302897 22427876

[b57] Terblanche J. S. . Ecologically relevant measures of tolerance to potentially lethal temperatures. J. Exp. Biol. 214, 3713–3725 (2011). 22031735 10.1242/jeb.061283

[b58] Lutterschmidt W. I. & Hutchison V. H. The critical thermal maximum: history and critique. Can. J. Zool. 75, 1561–1574 (1997).

[b59] Huey R., Crill W., Kingsolver J. G. & Weber K. A method for rapid measurement of heat or cold resistance of small insects. Funct. Ecol. 6, 489–494 (1992).

[b60] Hoffmann A. A., Sørensen J. G. & Loeschcke V. Adaptation of *Drosophila* to temperature extremes: bringing together quantitative and molecular approaches. J. Therm. Biol. 28, 175–216 (2003).

[b61] Chown S. L. & Terblanche J. S. Physiological diversity in insects: ecological and evolutionary contexts. Adv. In Insect Phys. 33, 50–152 (2006). 19212462 10.1016/S0065-2806(06)33002-0 PMC2638997

[b62] Gargano J. W., Martin I., Bhandari P. & Grotewiel M. S. Rapid iterative negative geotaxis (RING): a new method for assessing age-related locomotor decline in *Drosophila*. Exp. Gerontol. 40, 386–395 (2005). 15919590 10.1016/j.exger.2005.02.005

[b63] Nichols C. D., Becnel J. & Pandey U. B. Methods to assay *Drosophila* behavior. J. Vis. Exp. 61, 3795 (2012). 10.3791/3795 PMC3671839 22433384

[b64] Rasband W. S. ImageJ. U. S. National Institutes of Health (2014).

[b65] Allada R., Emery P., Takahashi J. S. & Rosbash M. Stopping time: the genetics of fly and mouse circadian clocks. Annu. Rev. Neurosci. 24, 1091–1119 (2001). 11520929 10.1146/annurev.neuro.24.1.1091

[b66] Bahrndorff S., Gertsen S., Pertoldi C. & Kristensen T. N. Investigating thermal acclimation effects before and after a cold shock in *Drosophila melanogaster* using behavioural assays. Biol. J. Linn. Soc. 117, 241–251 (2016).

[b67] Warnes G. R. . gplots: various R programming tools for plotting data. R package version 2.17.0 (2015).

[b68] R Core Team. R: a language and environment for statistical computing. R Foundation for Statistical Computing, Vienna, Austria. http://www.r-project.org/. (2016).

[b69] Bates D., Mächler M., Bolker B. M. & Walker S. C. Fitting linear mixed-effects models using lme4. J. Stat. Softw. 67, 1–48 (2015).

