# Supplementary material for: Biotic and abiotic factors investigated in two Drosophila species – evidence of both negative and positive effects of interactions on performance
Source: Sci Rep. 2017 Jan 6;7:40132. doi: 10.1038/srep40132 (PMC5216344; doi:10.1038/srep40132)
Supplement: Supplementary Information [file srep40132-s1.pdf]

Supplementary information for:

Biotic and abiotic factors investigated in two *Drosophila* species – evidence of both negative and positive effects of interactions on performance

Michael Ørsted[1*], Mads Fristrup Schou[2] and Torsten Nygaard Kristensen[1,2]

[1] Department of Chemistry and Bioscience, Section of Biology and Environmental Science, Aalborg University, Fredrik Bajers Vej 7H, DK-9220 Aalborg E, Denmark

[2] Department of Bioscience, Section of Genetics, Ecology and Evolution, Aarhus University, Ny Munkegade 114, DK-8000 Aarhus C, Denmark

* Corresponding author:

Michael Ørsted, email: moer@bio.aau.dk

Contents

Supplementary discussion 2

Supplementary Table S1 7

Supplementary Table S2 8

Supplementary Figure S1 9

Supplementary Figure S2 10

Supplementary Figure S3 11

Supplementary Figure S4 12

Supplementary Figure S5 14

Supplementary materials references 16

Supplementary discussion

The main point of this study is to present and discuss the results of a set of complex interactions in a multi-trait, multi-species analysis. In doing so, however, we realised certain problems with the classically defined synergism and antagonism terms. Others have discussed problems associated with the traditional definitions struggling to describe the situations of more complex outcomes, which seem to be fairly common when analysing interactions, both in laboratory experiments and in field studies[1–4]. Congruent with such studies, we point to challenges with the typical direction-independent classification because of the issues and limitations of the traditional framework outlined here. Also, a large numbers of studies on multiple environmental factors report interactions based on imprecise descriptions or simply the qualitative judgement of the authors[5]. Thus, in the scientific literature there is a lack of consensus on operationally robust definitions and quantification of synergism and antagonism[2,3,6–8].

The long-standing scientific classic definitions of synergism and antagonism are valid. We are merely proposing an expansion on the traditional definitions. As first proposed by Piggott et al. (2015)[4], and in our work expanded to include three-way interactions, we utilised a system combining the 'interaction effect' (as in the classic effect deviation from the additive model prediction[6]), with the magnitude and direction of the response (+ or –) relative to individual treatment effects in absolute terms. Thus, the "re-defined" synergism and antagonism still pertain to the classic "more than" and "less than", respectively, as it is traditionally understood.

The lack of consensus on definitions is most likely due to the usage of these terms throughout widely different scientific disciplines from ecology to toxicology and medicine. In toxicology and to some extent ecology (and thus ecotoxicology) interactions are frequently regarded as "stressful" and therefore exclusively detrimental to the overall performance of the subject species[4,6]. In this context of viewing interactions as always being negative, a synergistic interaction is defined as an

interaction causing negative effect greater than predicted by an additive model and an antagonistic interaction as a negative effect that is less than predicted from additivity.

To highlight the problems and limitations of the classic framework in ecological interactions, especially related to the "always-negative" view on interactions, we tried to re-designate classically defined terms to the interactions observed in our dataset (**Supplementary Figure 4** and **Supplementary Figure 5**). In doing so we identified several issues listed below, and compared our findings to other studies employing the traditional definition framework:

1) In situations where neither individual environmental condition has a significant effect on a trait but the interaction is significantly negative, interactions cannot be properly determined by classic definitions; e.g. when 0 + 0 < 0. Attempting to classify these situations in the classic paradigm would always result in synergism i.e. "more negative than" the individual effects, as antagonism is interpreted as "less negative than" the individual effects (not pertaining to the result being positive or negative in terms of fitness/performance, but how the interaction relates to the additive expectation). Examples from the present study that represent this sort of challenge in defining interactions in the classic framework are marked with a superscript "*x*" in **Supplementary Figure 4** and **Supplementary Figure 5**.
2) Even if still pertaining to the "all negative" nature of interactions, in situations where one individual factor has a negative effect while the other has no effect, and their cumulative effect is more negative than additively expected, the classic paradigm is also struggling. In (eco)toxicology this is sometimes referred to as potentiation or sensitisation[9] and some argue that it is not true "synergism" because it is one-sided and the underlying modes of action are different[10]. We have not included such further definitions, because it would confuse more than contribute, and we believe all situations are encompassed and informatively described by the re-conceptualised terms used in the present study.

Examples from the present study that represent this sort of challenge in defining interactions in the classic framework are marked with a superscript "*y*" in **Supplementary Figure 4** and **Supplementary Figure 5**.

In contrast to the persistent "all negative" view of interactions and the individual factors assessed, positive effects of individual environmental factors and even positive effects of interactions must be recognised. In ecotoxicology this phenomenon is often referred to as hormesis and is readily observed when assessing the effects of chemicals, e.g. at low dose[7,8,11]. While some "stressors" like chemicals are most frequently investigated as a gradient (concentrations), and thus might result in hormesis being observed at a low dose, other "stressors" are not as easily applied at a continuous scale e.g. biotic factors including co-occurring species or predation/parasitism, which is more of a presence/absence situation. Indeed, one could apply varying levels (densities) of co-occurring species or predators/parasites, but in a full-factorial study on interactions this would quickly scale to non-manageable proportions.

Even if accepting positive effects of interactions on performance or more importantly the positive effect (direction) of one or more individual factors, as employed in several recent reviews on interactions[1,4], we identify several issues using the classic definitions. While the identification of a synergism or antagonism is generally straightforward when all factors operate in the same direction[5,6], i.e. all positive (**Figure 1a**) or all negative (**Figure 1c**), problems arise when individual factors are of opposite directions (**Figure 1b**). In such situations the classic definition of synergism appears paradoxical because what is synergistic to the effect direction of one factor is antagonistic to the effect direction of the other factor(s):

3) By classic definitions it is difficult to classify interactions when the effects of two individual environmental conditions are in opposite direction e.g. $-1 + 1 > 1$ (see below for further discussion). Examples from the present study that represent this sort of challenge in

defining interactions in the classic framework are marked with a superscript "*z*" in **Supplementary Figure 4** and **Supplementary Figure 5**.

4) Crain et al. (2008)[1], having accepted the presence of positive effects, assumed that in situations with two opposing individual effect directions, synergy only occurred when the cumulative effect was more negative than the additive sum of the opposing individual effects. Examples from the present study where we have utilised this definition to define synergism are marked with a superscript "*w*" in **Supplementary Figure 4** and **Supplementary Figure 5**.

The definition of Crain et al. (2008)[1] may be appropriate if the effect direction is implicitly negative, e.g. decreased survival rate, but in many other situations such a definition is problematic from an ecological perspective because effect direction can be context dependent (see e.g. discussion of the effect direction on developmental time in main manuscript). This assumption raises another conceptual issue in that the cumulative effect of factors of opposing directions are not necessarily more negative than the single negative stressor acting alone (see "comparative effects" model of Folt et al. 1999[6]). Consider an example of a factor, which when applied alone has a positive effect of +1 and a factor, which when applied alone has a negative effect of –1. The additive cumulative effect of both factors combined is 0, i.e. they neutralise each other. By classic assumptions, as that of Crain et al. (2008)[1], we should invoke synergy for any cumulative effect more negative than 0. However, if the cumulative effect is between –1 and 0, this interaction is intuitively antagonistic from the perspective of the negative factor's individual effect (–1), i.e. the cumulative effect of both factors is less negative than the single negative stressor acting alone. In our proposed system, we would classify this as a *positive antagonism*, i.e. it is less positive than predicted from an additive model. Had the cumulative effect been between 0 and 1 we would classify it as a *negative antagonism*, i.e. it is less negative than predicted additively. Thus, antagonism can be easily interpreted in the traditional sense of "less than" in terms of the

cumulative effect relative to the effect of the individual effect size. The positive or negative prefix enables rapid interpretation of the direction relative to the cumulative effect, especially in these situations of opposing individual factors (**Figure 1b**). We want to re-enforce that these prefixes does not describe the performance or fitness effect of the interaction, i.e. a *positive antagonism* is not necessarily beneficial to the organism, partly because it can be difficult to establish the relationship between an effect direction and its costs and benefits to performance in some traits, e.g. as in the case of developmental time[12]. However this is not a problem specifically pertinent to our system, this is also a problem in the classic definitions framework.

The system also includes a new form of synergy, referred to as “mitigating synergism”, when individual environmental factors operating in the same direction interact and result in a cumulative effect in the opposite direction, e.g. two positives make a negative (S–) or two negatives make a positive (S+). Such strong interactions might be of great interest in predicting ecological consequences of multiple environmental factors, because different treatments can synergistically inhibit or mitigate the effect of individual factors[4,7].

While we realise that these introduced interaction terms can seem unduly complicated, we believe that the re-conceptualized terms provide more informative descriptions and straightforward interpretations of complex interactions, which would be difficult to even describe in the classic context.

Supplementary Table S1: Environmental treatments in a full factorial design, showing temperature, dimethoate concentration, co-occurrence status, number of vials in a respective treatment and number of eggs per vial. Temp.: temperature. Dim.: dimethoate. Co-occur.: co-occurrence. *D. hydei*: hyd. *D. melanogaster*: mel.

Identification code	Species	Temp. (°C)	Dim. (ppb)	Co-occur.	Number of vials	Number of eggs per vial
h-25-0	hyd	25	0	No	30	40
h-25-75	hyd	25	75	No	40	40
m-25-0	mel	25	0	No	30	40
m-25-75	mel	25	75	No	30	40
h/m -25-0	hyd/mel	25	0	Yes	30	20 of each species
h/m -25-75	hyd/mel	25	75	Yes	40	20 of each species
h-13-0	hyd	13	0	No	30	40
h-13-75	hyd	13	75	No	40	40
m-13-0	mel	13	0	No	30	40
m-13-75	mel	13	75	No	30	40
h/m-13-0	hyd/mel	13	0	Yes	30	20 of each species
h/m -13-75	hyd/mel	13	75	Yes	40	20 of each species
h-31-0	hyd	31	0	No	30	40
h-31-75	hyd	31	75	No	40	40
m-31-0	mel	31	0	No	30	40
m-31-75	mel	31	75	No	30	40
h/m -31-0	hyd/mel	31	0	Yes	30	20 of each species
h/m -31-75	hyd/mel	31	75	Yes	40	20 of each species

Supplementary Table S2: Number of flies used from each species from each treatment for CT_{min}, CT_{max}, developmental time, and RING for each sex and for egg-to-adult viability for both sexes combined. Average number of flies (and standard deviation (S.D.)) from each trait is also given. NA values indicate that no or too few flies emerged from a given treatment. The minimum number of measurements (limit *n*) for CT_{min}, CT_{max}, and developmental time was 5 and for RING and egg-to-adult viability it was 50 and 30, respectively. Dim.: dimethoate. Co-occur.: co-occurrence.

	**Trait**	**CT_{max}**		**CT_{min}**		**Developmental time**		**RING**		**Viability**
	**Sex**	**Males**	**Females**	**Males**	**Females**	**Males**	**Females**	**Males**	**Females**	**Both**
D. hydei	25 °C (Control)	8	17	9	20	119	116	100	100	30
	25 °C + Co-occur.	20	22	26	28	145	163	100	100	30
	25 °C + Dim.	21	19	30	20	162	129	100	100	30
	25 °C + Co-occur. + Dim.	19	17	27	26	68	65	100	100	40
	13 °C	NA	7	10	12	182	199	100	100	30
	13 °C + Co-occur.	22	16	13	16	172	137	100	50	30
	13 °C + Dim.	10	12	14	15	43	35	50	NA	30
	13 °C + Co-occur. + Dim.	NA	NA	NA	NA	NA	6	NA	NA	40
	31 °C	9	10	9	9	66	60	NA	100	30
	31 °C + Co-occur.	6	12	7	14	40	49	50	50	30
	31 °C + Dim.	10	8	10	8	46	54	100	50	30
	31 °C + Co-occur. + Dim.	20	20	19	19	134	102	100	100	40
	Average *n*	15	15	16	17	107	93	90	85	33
	S.D.	6	5	8	6	53	55	20	23	4
D. melanogaster	25 °C (Control)	20	19	20	20	466	494	100	100	30
	25 °C + Co-occur.	19	18	20	20	276	262	100	100	30
	25 °C + Dim.	19	19	19	20	424	434	100	100	30
	25 °C + Co-occur. + Dim.	18	17	20	18	354	368	100	100	40
	13 °C	27	20	18	20	440	516	100	100	30
	13 °C + Co-occur.	19	20	20	20	208	209	50	100	30
	13 °C + Dim.	20	20	19	20	439	428	100	100	30
	13 °C + Co-occur. + Dim.	20	20	19	20	306	313	100	100	40
	31 °C	19	20	20	20	475	491	100	100	30
	31 °C + Co-occur.	19	20	19	20	218	233	100	100	30
	31 °C + Dim.	19	20	20	20	492	495	100	100	30
	31 °C + Co-occur. + Dim.	19	20	20	19	301	326	100	100	40
	Average *n*	20	19	20	20	367	381	96	100	33
	S.D.	2	1	1	1	98	106	14	0	4
	Limit *n*	5	5	5	5	5	5	50	50	30

Supplementary Figure S1

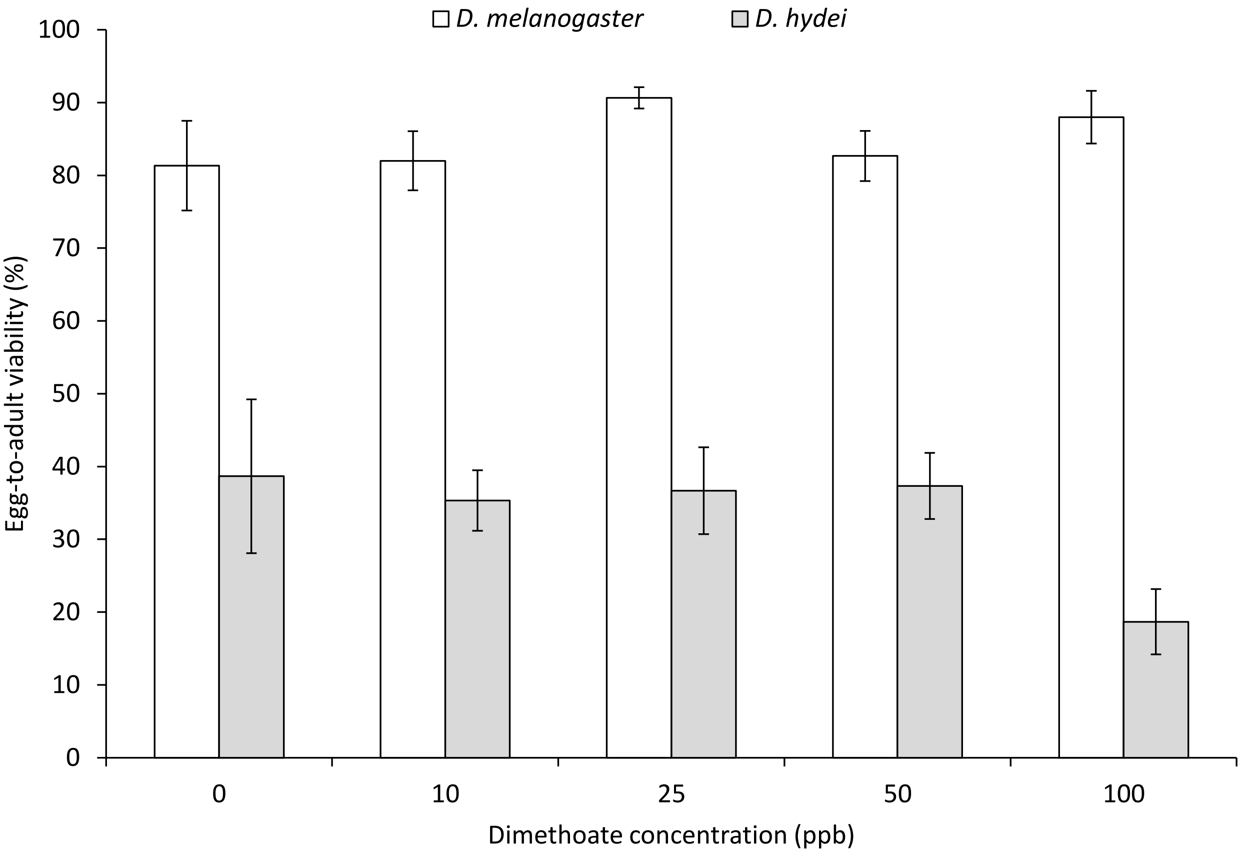

Supplementary Figure S1: Preliminary screening of egg-to-adult viability (%) in *D. hydei* (grey filled bars) and *D. melanogaster* (white open bars) at a range of dimethoate concentration from 0 to 100 ppb. Error bars represent standard error (n = 5). The media setup, egg collection procedure and subsequent scoring of viability followed the same procedure as described in the main experiment (40 eggs per vial with 9 mL Formula 4-24® Instant *Drosophila* Medium Blue ± dimethoate).

Supplementary Figure S2

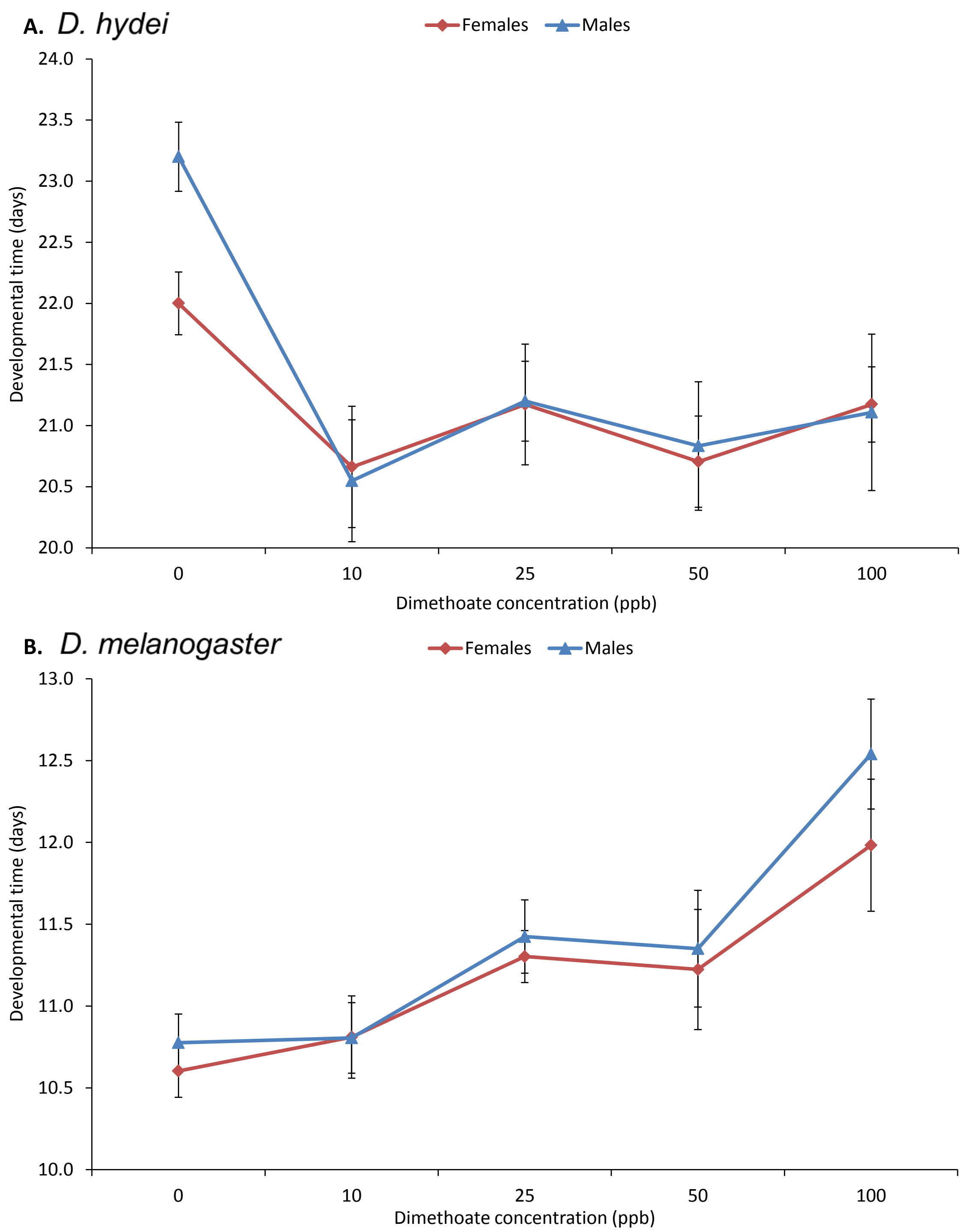

Supplementary Figure S2: Preliminary screening of egg-to-adult developmental time (in days) in females (red) and males (blue) of *D. hydei* (**A.**) and *D. melanogaster* (**B.**) at a range of dimethoate concentration from 0 to 100 ppb. Error bars represent standard error (n = 5). The media setup, egg collection procedure and subsequent scoring of developmental time followed the same procedure as described in the main experiment (40 eggs per vial with 9 mL Formula 4-24® Instant *Drosophila* Medium Blue ± dimethoate).

Supplementary Figure S3

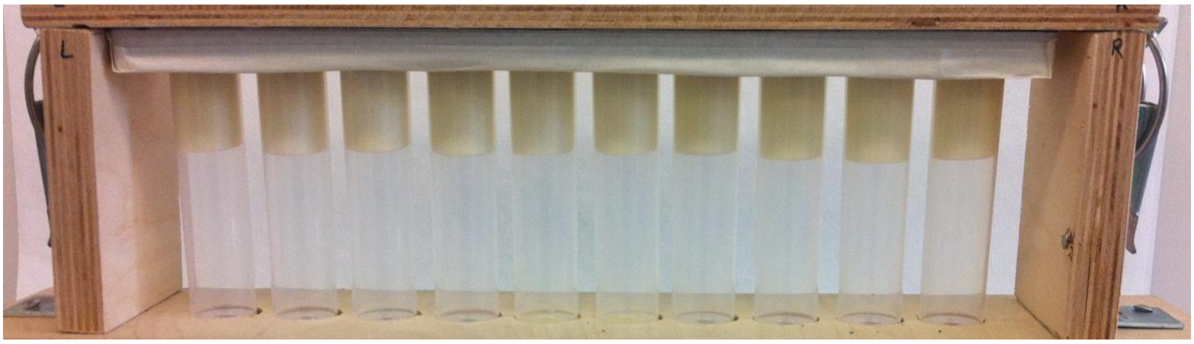

Supplementary Figure S3: Front side view of the Rapid Iterative Negative Geotaxis (RING) assay apparatus modified from Gargano et al. (2005)[13]. Our version of the RING apparatus was a custom built open-faced wooden box with a detachable lid held in place with lock hinges. The rack holds 10 empty vertical 27 mL vials. Into the floor of the apparatus was milled a 1 mm indentation as support for 10 empty vertical 27 mL vials. Foam stoppers were inserted to an equal depth across all 10 vials and the lid of the apparatus holds the negative geotaxis vials in place when locked with hinges. A camera was mounted 30 cm from the apparatus to capture photos of the negative geotaxis behaviour.

Supplementary Figure S4

Drosophila hydei

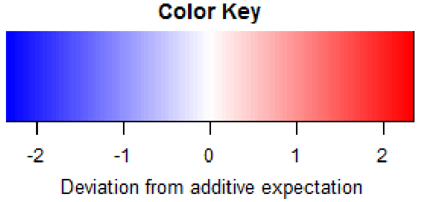

Trait and sex

	CT$_{max}$ Females	CT$_{max}$ Males	CT$_{min}$ Females	CT$_{min}$ Males	Developmental time Females	Developmental time Males	Negative geotaxis Females	Negative geotaxis Males	Egg-to-adult viability	Total average		
Interactions	0.55 * S	0.86	0.32	-0.55	0.17 * S	0.2 * S	1.45	NA	2.35 * $?^{z}$	0.67 ± 0.32	Heat x Co-occur. x Dim.
Interactions	-1.18 * A^{y}	NA	-0.08	-0.31	-0.07 * S^{y}	-0.15 * $S^{z,w}$	-0.02	0.67	0.15	-0.12 ± 0.18	Cold x Co-occur.
Interactions	-0.4 * A^{y}	-1.36 * A^{y}	-0.44	0.31	-0.17 * A	-0.19 * A	-0.37	NA	0.19	-0.3 ± 0.18	Heat x Dim.
Interactions	-0.64 * A	-0.75	0.02	0.4	-0.16 * A	-0.24 * A	-1.13	NA	-0.28	-0.35 ± 0.17	Heat x Co-occur.
Interactions	-0.14	-0.64	-0.19	-0.01	-0.03	-0.08	-1.21	NA	-1.22 * $S^{z,w}$	-0.44 ± 0.18	Co-occur. x Dim.
Interactions	-0.62	NA	-0.27	-0.27	0.1 * $?^{z}$	0.05	NA	-0.7	-1.46 * S^{y}	-0.45 ± 0.20	Cold x Dim.
Interactions	NA	NA	NA	NA	-0.05	NA	NA	NA	-1.31 * $S^{z,w}$	-0.68 ± 0.63	Cold x Co-occur. x Dim.
Individual factors	0.49 *	1.03 *	0.11	-0.1	0.12 *	0.19 *	1.38 *	1.32 *	0.67 *	0.58 ± 0.18	Co-occur.
Individual factors	0.2	0.76	0.45 *	-0.02	0.11 *	0.13 *	1.11 *	1.12 *	-0.44 *	0.38 ± 0.18	Dim.
Individual factors	0.59	NA	1.96 *	2.14 *	-1.28 *	-1.24 *	-0.9 *	-0.9 *	-0.14	0.03 ± 0.49	Cold
Individual factors	0.86 *	2.27 *	-1.68 *	-2.07 *	0.54 *	0.6 *	-0.67 *	NA	-1.52 *	-0.21 ± 0.54	Heat

Supplementary Figure S4: Heat map showing the direction and magnitude of the model coefficients reflecting the effects of treatments on egg-to-adult viability, developmental time, CT_{min}, CT_{max} and negative geotaxis (RING assay) in *D. hydei*. The effects are shown for both sexes in all traits except egg-to-adult viability. Positive coefficients represent positive deviation from the additive expectation, and can thus be interpreted as a performance advantage of the interaction itself, regardless of whether the treatment overall was beneficial in terms of performance when compared to the control. Contrary, a negative coefficient implies a negative deviation from additivity and that the interaction itself is detrimental to performance for a given trait. The direction of the effect is illustrated by colour shading from blue (negative) to red (positive) and the values indicate the strength of the effects. The upper part includes all two- and three-way interactions between heat or cold, co-occurrence (Co-occur.), and dimethoate (Dim.). The lower part includes all effects of the individual factors. Within each part the treatments (rows) have been sorted by the average total effect, i.e. the average effect across traits ± S.E., in descending order. An asterisk indicates a significant interaction, or a significant effect of the individual environmental factor. The direction of the interaction has been determined based on the traditional definitions of synergism (S) and antagonism (A). In doing so we identified several issues; the nature of these challenges is marked with subscripts $^{w, x, y}$ or ^{z} next to the designation of the interaction. Some interactions might relate to several issues and are given multiple subscript characters, and some interactions simply could not be determined based on contradicting definitions of the classic framework (designated with a question mark). The details of these challenges are described in the text in the **Supplementary discussion**. Some treatments did not yield enough live adult flies for assessing all traits or did not exceed the minimum number of flies needed for assessing a trait. In a few traits the effect of an individual environmental factor could therefore not be determined, and the interactions involving the particular factor were omitted from the model. Both cases are designated NA.

Supplementary Figure S5

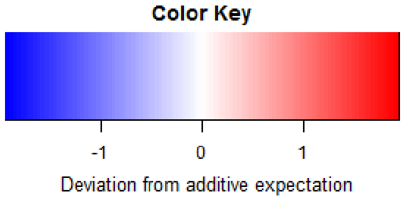

Drosophila melanogaster

Trait and sex

		CTmax Females	CTmax Males	CTmin Females	CTmin Males	Developmental time Females	Developmental time Males	Negative geotaxis Females	Negative geotaxis Males	Egg-to-adult viability	Total average
Interactions	Co-occur. x Dim.	0.01	0.4	0.03	0.19	-0.02	0.11 * A^{y}	0.54	-0.33	0.98 * A^{x}	0.21 ± 0.13
	Cold x Co-occur.	0.33	0.61 * A^{y}	-0.08	0.05	-0.02	0.12 * A	0.36	-0.14	-0.8 * S^{x}	0.05 ± 0.13
	Heat x Dim.	-0.19	0.34	0.09	-0.26	0	0.02	0.28	-0.55	0.64 * A^{x}	0.04 ± 0.12
	Cold x Dim.	-0.37	0.22	-0.01	0.08	-0.06 * S^{y}	-0.02	0.66 * A	-0.32	0.12	0.03 ± 0.10
	Heat x Co-occur. x Dim.	0.33	-0.46	-0.31	0.4	0.12 * S^{y}	-0.02	-0.73	-0.05	-0.92	-0.18 ± 0.15
	Cold x Co-occur. x Dim.	0.2	-0.69	0.1	-0.24	0.11 * A^{y}	-0.08	-1.47 * $S^{z,w}$	0.13	-0.18	-0.24 ± 0.18
	Heat x Co-occur.	0.42	-0.4	-0.36	-0.22	-0.09 * A^{y}	0.03	-1.06	-0.15	-0.58	-0.27 ± 0.14
Individual factors	Co-occur.	-0.24	-0.11	0.39	0.3	0.04	-0.09 *	0.24 *	0.13	0.26	0.1 ± 0.07
	Heat	1.19 *	0.5	-1.19 *	-1.37 *	0.28 *	0.27 *	0.03	-0.4 *	0.03	-0.07 ± 0.27
	Dim.	0.09	-0.13	-0.26	0.01	0.01	0	-0.34 *	0.23 *	-0.53	-0.1 ± 0.08
	Cold	-1.69 *	-1.82 *	1.94 *	1.87 *	-1.38 *	-1.42 *	-0.51 *	-0.9 *	-0.02	-0.44 ± 0.48

Supplementary Figure S5: Heat map showing the direction and magnitude of the model coefficients reflecting the effects of treatments on egg-to-adult viability, developmental time, CT_{min}, CT_{max} and negative geotaxis (RING assay) in *D. melanogaster*. The effects are shown for both sexes in all traits except egg-to-adult viability. Positive coefficients represent positive deviation from the additive expectation, and can thus be interpreted as a performance advantage of the interaction itself, regardless of whether the treatment overall was beneficial in terms of performance when compared to the control. Contrary, a negative coefficient implies a negative deviation from additivity and that the interaction itself is detrimental to fitness. The direction of the effect is illustrated by colour shading from blue (negative) to red (positive) and the values indicate the strength of the effects. The upper part includes all two- and three-way interactions between heat or cold, co-occurrence (Co-occur.), and dimethoate (Dim.). The lower part includes all effects of the individual factors. Within each part the treatments (rows) have been sorted by the average total effect, i.e. the average effect across traits ± S.E., in descending order. An asterisk indicates a significant interaction, or a significant effect of the individual environmental factor. The direction of the interaction has been determined based on the traditional definitions of synergism (S) and antagonism (A). In doing so we identified several issues; the nature of these challenges is marked with subscripts $^{w,\, x,\, y}$ or ^{z} next to the designation of the interaction. Some interactions might relate to several issues and are given multiple subscript characters. The details of these challenges are described in the text in the **Supplementary discussion**.

Supplementary materials references

1. Crain, C. M., Kroeker, K. & Halpern, B. S. Interactive and cumulative effects of multiple human stressors in marine systems. *Ecol. Lett.* **11,** 1304–1315 (2008).

2. Darling, E. S. & Côté, I. M. Quantifying the evidence for ecological synergies. *Ecol. Lett.* **11,** 1278–1286 (2008).

3. Vanhoudt, N., Vandenhove, H., Real, A., Bradshaw, C. & Stark, K. A review of multiple stressor studies that include ionising radiation. *Environ. Pollut.* **168,** 177–192 (2012).

4. Piggott, J. J., Townsend, C. R. & Matthaei, C. D. Reconceptualizing synergism and antagonism among multiple stressors. *Ecol. Evol.* **5,** 1538–1547 (2015).

5. Dunne, R. P. Synergy or antagonism-interactions between stressors on coral reefs. *Coral Reefs* **29,** 145–152 (2010).

6. Folt, C., Chen, C., Moore, M. & Burnaford, J. Synergism and antagonism among multiple stressors. *Limnol. Oceanogr.* **44,** 864–877 (1999).

7. Holmstrup, M. *et al.* Interactions between effects of environmental chemicals and natural stressors: a review. *Sci. Total Environ.* **408,** 3746–3762 (2010).

8. Laskowski, R. *et al.* Interactions between toxic chemicals and natural environmental factors - a meta-analysis and case studies. *Sci. Total Environ.* **408,** 3763–3774 (2010).

9. Odum, E. P. & Barrett, G. W. *Fundamentals of Ecology*. (Thomson/Brooks/Cole, Belmont, CA, USA, 2005).

10. Chou, T. C. Drug combination studies and their synergy quantification using the Chou-Talalay method. *Cancer Res.* **70,** 440–446 (2010).

11. Boonstra, R., Manzon, R. G., Mihok, S. & Helson, J. E. Hormetic effects of gamma radiation on the stress axis of natural populations of meadow voles (*Microtus pennsylvanicus*). *Environ. Toxicol. Chem.* **24,** 334–343 (2005).

12. Niemelä, P. T., Vainikka, A., Hedrick, A. V. & Kortet, R. Integrating behaviour with life history: boldness of the field cricket, *Gryllus integer*, during ontogeny. *Funct. Ecol.* **26,** 450–456 (2012).

13. Gargano, J. W., Martin, I., Bhandari, P. & Grotewiel, M. S. Rapid iterative negative geotaxis (RING): A new method for assessing age-related locomotor decline in *Drosophila*. *Exp. Gerontol.* **40,** 386–395 (2005).
